# Chiral‐Nematic Lattice Instability in Charge Density Wave of 1*T*‐TiSe_2_

**DOI:** 10.1002/advs.78243

**Published:** 2026-10-20

**Authors:** Abdurrafi Ahsan Alghifari, Euihwan Do, Jae Whan Park, Han Woong Yeom

**Affiliations:** ^1^ Center for Artificial Low Dimensional Electronic Systems Institute for Basic Science (IBS) Pohang Republic of Korea; ^2^ Department of Physics Pohang University of Science and Technology (POSTECH) Pohang Republic of Korea

**Keywords:** 1*T*‐TiSe_2_, charge density wave, chiral symmetry breaking, density functional theory, nematic fluctuations, scanning tunneling microscopy

## Abstract

Chiral charge‐density waves (CDWs) are a rare form of electronic order with handedness, enabling novel couplings among light, lattice, and topology in quantum materials. Yet the microscopic origin of chirality has remained elusive, even in the prototypical chiral CDW system 1*T*‐TiSe_2_. Using ab initio calculations, we investigate the structural stability and electronic structure of competing CDW models in 1*T*‐TiSe_2_. We show that a chiral monoclinic (C2) structure is stabilized by the cooperative condensation of multiple phonon modes and reproduces the chiral topography and energy gap observed experimentally. We further find an exceptionally soft potential landscape around this energy‐minimized structure, along the direction that removes the remaining rotational symmetry, allowing the lattice to fluctuate through fully asymmetric configurations while its average structure remains chiral. This dynamical chiral CDW naturally reconciles the chiral domains seen by time‐averaged topographic probes with the complete loss of symmetry detected by bulk spectroscopy, and clarifies how chiral CDWs emerge from the interplay of lattice instability and fluctuation.

## Graphical abstract

The charge density wave of 1*T*‐TiSe_2_ appears chiral to surface probes yet loses all symmetry in spectroscopic measurements. This study reveals how cooperative condensation of multiple phonon modes stabilizes a chiral monoclinic ground state, while a shallow nematic energy landscape allows the lattice to break the remaining symmetry at negligible cost, reconciling two seemingly contradictory experiments.

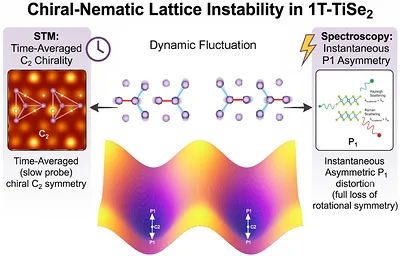

**Abbreviations**
ARPES: angle‐resolved photoemission spectroscopy
CDW: charge density wave
DFT: density functional theory
LDOS: local density of states
STM: scanning tunneling microscopy
STS: scanning tunneling spectroscopy

## 1 Introduction

Charge density waves (CDWs) are a fundamental way that electrons and lattices couple to lower the total energy of a system through spontaneously breaking its translational symmetry [1, 2]. CDWs are also important because they are often related to other exotic electronic properties as in the case of their competition or coexistence with superconductivity [3, 4, 5]. Recent progress in the investigation of CDWs has revealed extra symmetry breaking within CDW states, most notably, the formation of chiral CDW phases with broken mirror and inversion symmetry [6, 7, 8, 9, 10, 11]. Chiral CDWs can couple to topological degrees of freedom [12], chiral light polarizations [13, 14], and possibly to chiral phonons [15]. The helicity‐dependent dynamics of the chiral CDW is presently under active debate [14, 16, 17]. This phenomenon has recently been observed in a wider class of materials such as Kagome superconductors [18], where the chiral CDW is related to the time‐reversal symmetry breaking in the coexisting superconducting state. However, the driving forces of such extra symmetry breaking have been challenging to identify in many cases.

A model system for investigating this phenomenon has been 1*T*‐TiSe_2_, where the chiral CDW was discovered first [6]. 1*T*‐TiSe_2_ hosts a commensurate 2×2×2 CDW modulation with a transition temperature $T_{c}\approx 200$ K [19]. Suppressing this CDW order through copper intercalation or applied pressure induces a superconducting phase that peaks near the CDW critical point [3, 4, 5]. The chiral distortion in this CDW state was first identified [6] and established [20] by scanning tunneling microscopy, and recently confirmed by the circular photogalvanic effect [13] and angle‐resolved photoelectron spectroscopy [21]. However, despite extensive investigation, the microscopic structural model of the chiral distortion is still elusive [22], leaving the origin of inversion‐symmetry breaking and its connection to the electronic ground state unclear.

In particular, most theoretical investigations so far share the assumption that the low‐temperature phase adopts the centrosymmetric $P\bar{3}c1$ structure identified in early structural studies [19, 23]. This achiral phase is obviously incompatible with the observation of chiral domains in STM topography [20, 24] and the chiral response in circular photogalvanic measurements [13]. Furthermore, standard DFT calculations using this model typically predict a metallic (or semimetallic) band structure, requiring the inclusion of strong excitonic interaction [25, 26, 27, 28] or on‐site Coulomb interaction to reproduce the experimentally observed CDW gap of about 140 meV [29, 30, 31]. In contrast, a few recent experimental [32, 33] and theoretical [34, 35] studies have strengthened the case for a lattice origin, building on earlier evidence for the interplay of excitonic and phononic contributions [36].

In an effort to solve this problem, recent DFT calculations showed that a monoclinic C2 chiral CDW structure can emerge from the interlayer shear displacement of the $P\bar{3}c1$ structure [37], which matches well the chiral STM topography [20, 38]. Two subsequent studies have independently converged on the same chiral C2 arrangement, through a systematic enumeration of interlayer CDW phases [39] and through an interlayer stacking‐order analysis [40]. On the other hand, a recent spectroscopic study combined with theoretical modeling [41] suggested that a competition between different CDW components induced by charge‐lattice symmetry frustration can result in a chiral CDW distortion. This work, however, assumed the electronic order as the primary source of the instability and did not explicitly suggest any atomic structure for the chiral distortion. Surprisingly, this “charge‐lattice frustration” breaks all mirror and inversion symmetries so that the chiral CDW phase has no explicit crystal symmetry (P1) without a sign of the C2 symmetry. This discrepancy leaves the origin of chiral CDW in this model system elusive.

In the present work, we resolve this apparent discrepancy by mapping the full energy landscape of the competing distortion modes. Building upon prior phonon surveys that identified the C2 structure as a harmonic minimum [37], our ab initio analysis establishes that this chiral phase is a sufficient lattice‐driven model to explain most of the experimental observations. It reproduces the chiral topography, the energy gap, and the domain‐wall structures [20]. Furthermore, we discover that this C2 ground state sits at the center of a soft nematic energy manifold, where the remaining rotational symmetry can be removed at a minimal energetic cost. The lattice fluctuates through fully asymmetric configurations while its average structure remains chiral. This dynamic fluctuation reconciles the experimental paradox; fast spectroscopic probes detect the instantaneous P1 symmetry loss, while temporally‐integrating topographic probes capture the spatially averaged C2 chirality. Consequently, the present work provides a lattice‐driven mechanism of chiral CDW without invoking static phase separation [41] or assuming a frustration between charge and lattice degrees of freedom [41].

## 2 Results

### 2.1 Phonon Modes and Possible Structure Models

To map the energy landscape of possible lattice distortions, we start from the primary lattice instabilities of the parent phase, that is, the L‐point and M‐point transverse optical modes hybridizing with the acoustic branches, which were identified in previous phonon studies [37, 42, 43]. The L‐mode corresponds to transverse in‐plane displacements driving Ti‐trimer formation with anti‐phase interlayer stacking ($k_{z}=0.5$), while the M‐mode corresponds to in‐phase stacking ($k_{z}=0$) (Figure 1). We construct candidate structures by combining these L and M distortions across one, two, or all three propagation directions ($q_{1},q_{2},q_{3}$). This generates a complete hierarchy of candidate phases spanning various symmetries, as detailed in Table 1.

**TABLE 1:** Energetic hierarchy and symmetry classification of the structural phases derived from M‐point ($\Gamma_{5}^{3}$) and L‐point ($\Gamma_{7}^{3}$) phonon modes. Energies (ΔE) are given in meV per supercell relative to the high‐symmetry parent phase.

Family	Distortion	Symmetry	ΔE (meV/cell)
Reference	—	$P\bar{3}m1$	0.00
Pure $\Gamma_{5}^{3}$ (M)	$1Q_{M}$	P2/c	−9.7
	$2Q_{M}$	C2/m	−13.4
	$3Q_{M}$	P321	−15.5
Pure $\Gamma_{7}^{3}$ (L)	$1Q_{L}$	C2/c	−10.7
	$2Q_{L}$	C2/m	−14.9
	$3Q_{L}$	$P\bar{3}c1$	−17.2
Mixed (L+M)	$1Q_{M}+1Q_{L}$	$P\bar{1}$	−14.5
	$2Q_{M}+1Q_{L}$	C2/c	−17.7
	**2Q_L_ + 1Q_M_**	**C2**	**−18.8**

**FIGURE 1:**
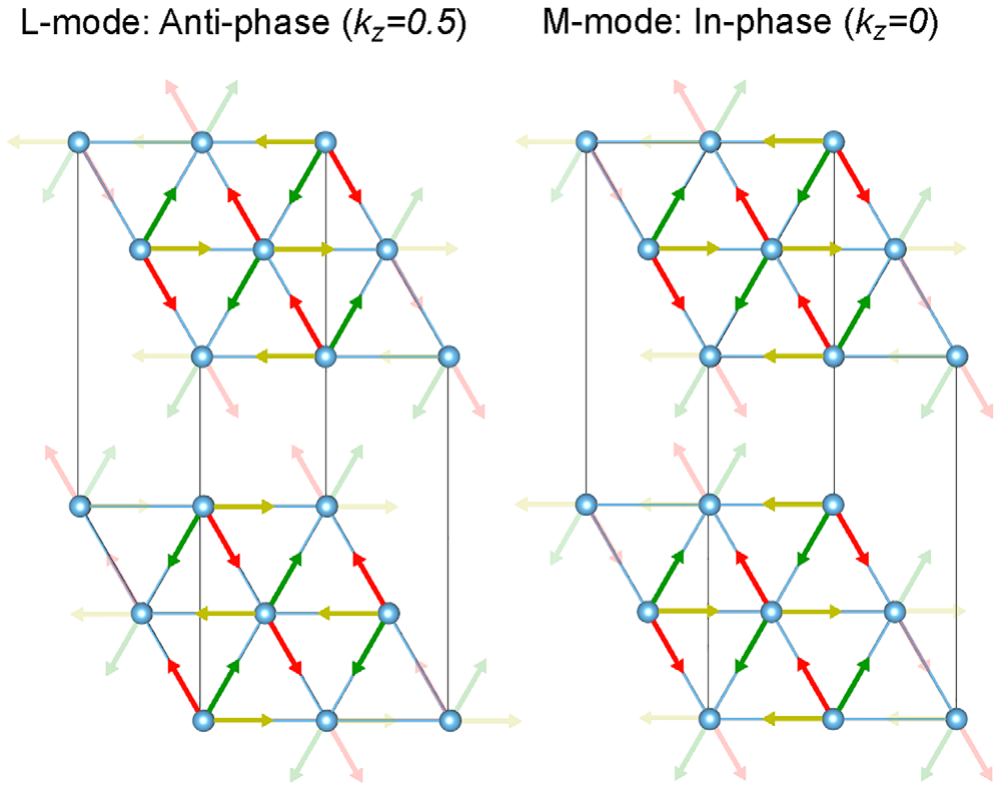
Phonon eigenvectors and stacking configurations of the primary lattice instabilities. **(Left)** The L‐point transverse optical phonon mode, characterized by anti‐phase interlayer stacking ($k_{z}=0.5$), drives Ti‐trimer formation within each layer through transverse in‐plane displacements. **(Right)** The M‐point mode exhibits in‐phase stacking ($k_{z}=0$) and describes uniform interlayer shear. The color‐coded arrows indicate displacement directions for the three propagation vectors ($q_{1}$, $q_{2}$, $q_{3}$) of the 2×2×2 supercell. The directional selection and cooperative mixing of these modes ($2Q_{L}+1Q_{M}$) stabilize the chiral C2 ground state. Se atoms are omitted for clarity.

To understand how these modes compete and mix, we introduce a Landau free‐energy expansion. The total free energy $F=F_{L}+F_{M}+F_{int}$ is constructed from energetic contributions from individual modes and the interactions between them. The leading interaction ($F_{int}$) between the L‐ and M‐mode amplitudes on the i‐th propagation axis ($Q_{L,i}$ and $Q_{M,i}$) is governed by fourth‐order interaction terms:

(1) $$F_{\text{int}}=\kappa \sum_{i}Q_{L,i}^{2}Q_{M,i}^{2}+\gamma \sum_{i\neq j}Q_{L,i}^{2}Q_{M,j}^{2}.$$

Fitting our ab initio total energies to this expansion yields κ>γ>0. The large positive κ represents a severe energetic penalty for simultaneous condensation of both L and M‐mode distortions on the same axis. This is simply because the anti‐phase (L‐mode) and in‐phase (M‐mode) trimerizations compete for the same in‐plane coordinate. To minimize the penalty while maximizing the condensation energy of both modes, the lattice strictly avoids mixed modes on any single axis. The term γ governs the weaker cross‐axis coupling. Because our calculated coefficients show the system energetically favors L‐mode condensation over the M‐mode (See Supporting Information), it selects an asymmetric configuration of anti‐phase trimerization on two axes and in‐phase on the third ($2Q_{L}+1Q_{M}$). This specific configuration eliminates the κ penalty entirely and co‐aligns the Ti‐trimers along a single crystallographic axis. By breaking both three‐fold rotational and inversion symmetries, this cooperative condensation naturally establishes the chiral C2 structure as the lowest‐energy state (Figure 3b and Table 1). Although another low energy structure (Table 1 and Figure 2a), C2/c, exists with a proximal energy difference, a transition from C2 to C2/c is highly unfavorable: it would require completely suppressing the distortion along one axis before rebuilding it on another. The corresponding energy barrier is equivalent to the energy needed to melt the distortion along an entire direction, preventing such a transition and trapping the system in the C2 energy‐minimum structure upon cooling.

**FIGURE 2:**
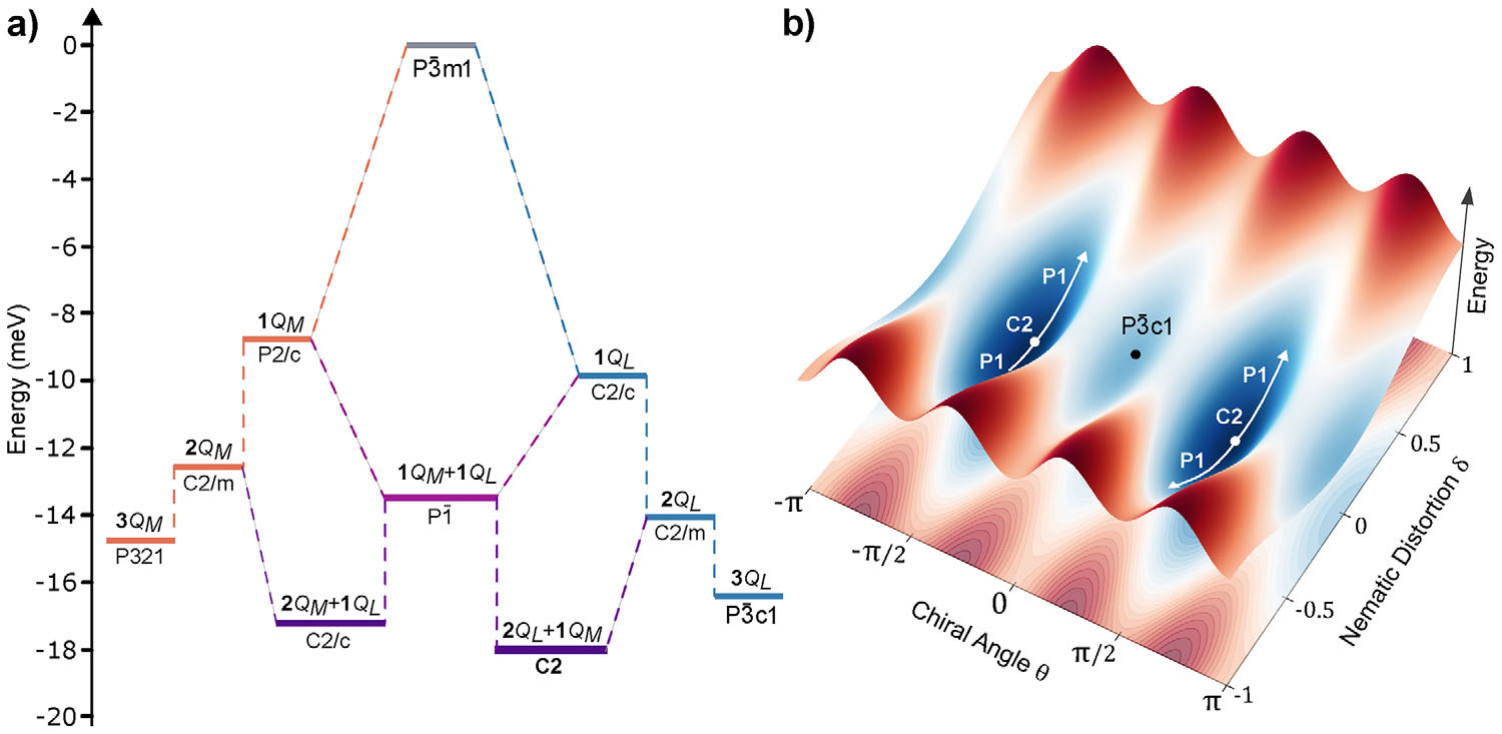
Ab initio energetic hierarchy and the chiral‐nematic energy surface. (a) Condensation energy diagram for structural phases derived from M‐point (orange) and L‐point (blue) lattice instabilities, relative to the $P\bar{3}m1$ parent phase. The cooperative $2Q_{L}+1Q_{M}$ condensation (purple) establishes the chiral C2 configuration as the global energetic minimum, separated from the canonical achiral $P\bar{3}c1$ ($3Q_{L}$) state by an activation barrier. (b) Potential energy landscape parameterized by the chiral mixing angle (θ) and the transverse nematic distortion (δ). The topology exhibits a double‐well structure along θ, separating left‐ and right‐handed C2 ground states (θ=±π/2) by the $P\bar{3}c1$ saddle point (θ=0). Along the δ‐axis, the landscape exhibits a flat soft manifold (white arrows), indicating the accessibility of triclinic P1 configurations at low energetic cost.

**FIGURE 3:**
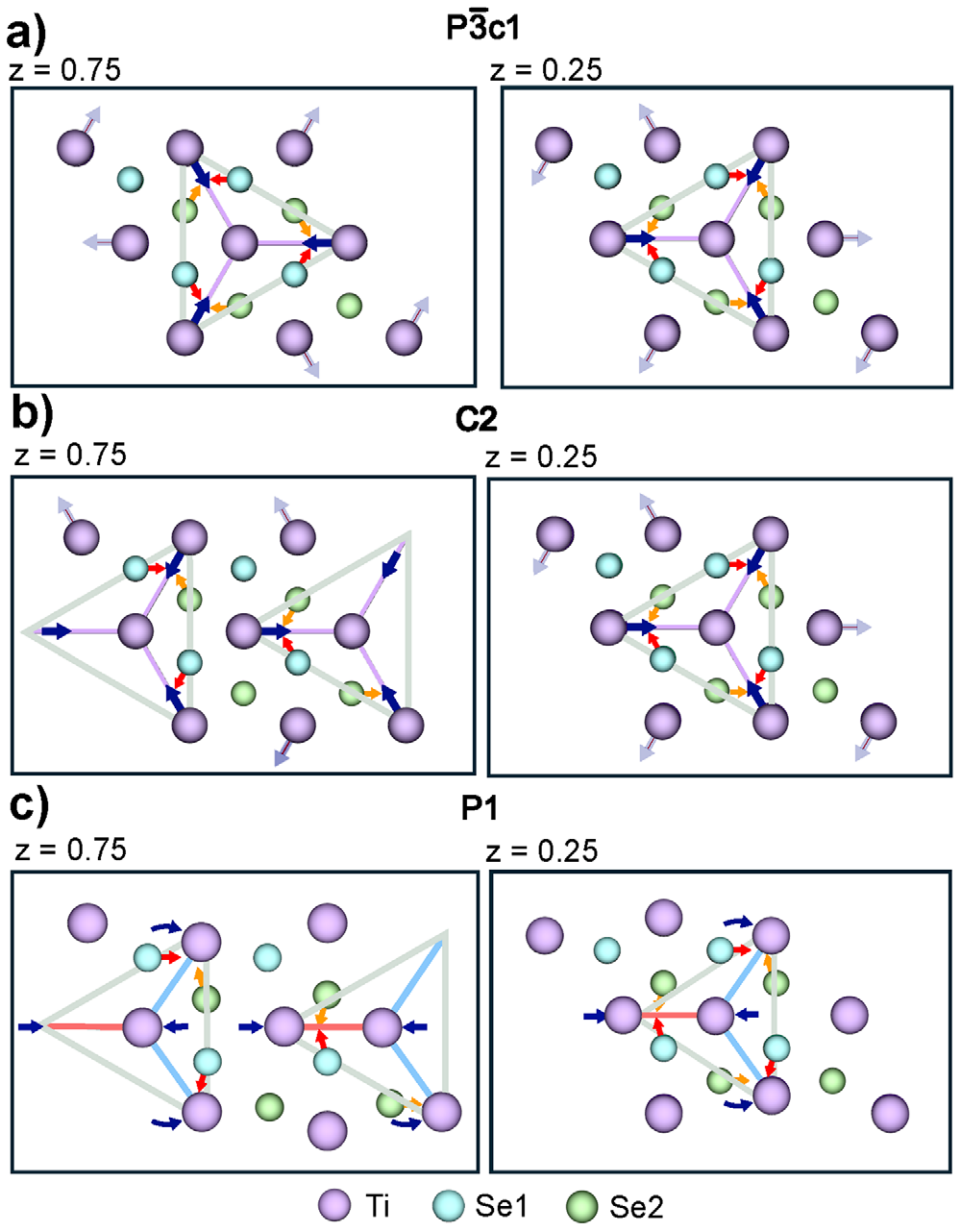
Real‐space structural decomposition of the charge density wave models. Top‐down view of the 2D projected atomic displacements for Ti layers at z=0.75 and z=0.25. Ti atoms are shown in purple, Se atoms in the top layer (Se1) in blue, and Se atoms in the bottom layer (Se2) in green. (a) The canonical achiral $P\bar{3}c1$ state driven by an isotropic $3Q_{L}$ condensation, featuring symmetric Ti‐trimer formations that preserve three‐fold rotational and inversion symmetries. (b) The chiral C2 ground state driven by the $2Q_{L}+1Q_{M}$ directional selection. The superposition of anti‐phase trimerization (L‐mode) and uniform interlayer shear (M‐mode) establishes a unique crystallographic axis and co‐aligns the Ti‐trimers, explicitly breaking both three‐fold rotational and inversion symmetries. (c) The triclinic P1 phase resulting from nematic fluctuations along the soft δ‐coordinate. Amplitude imbalance between the two active L‐modes induces intra‐trimer bond disproportionation, breaking the remaining two‐fold rotation axis, as highlighted by the contracted (red) and stretched (blue) Ti–Ti bonds.

The relative sign of the $2Q_{L}$ and $1Q_{M}$ amplitudes dictates the specific chirality (right‐ vs. left‐handedness) of the C2 structures. To see how these structures are connected, we map the energy landscape using two collective coordinates (Figure 2b). The first is the chiral mixing angle (θ). It describes the distortion path that flips the system's handedness. Interestingly, the shortest path between these C2 states passes through the achiral $P\bar{3}c1$ structure, which serves as the saddle point. The second is the nematic distortion (δ), defined mathematically as the amplitude imbalance between the two active transverse L‐modes. Physically, this distortion manifests as the unequal stretching and compressing of bonds within the Ti‐trimers. This bond‐stretching distortion breaks the remaining two‐fold rotational symmetry of the monoclinic lattice, resulting in a fully asymmetric, triclinic P1 configuration. This coordinate space describes only continuous local distortions within the $2Q_{L}+1Q_{M}$ framework; reaching the other phases in Table 1 would require discontinuously switching the modes to different propagation axes.

Crucially, our calculations reveal that the energy landscape is extremely flat along the bond‐stretching δ coordinate, yet with a single minimum at C2 (Figure 2b). Displacements along δ cost an energy of 1 meV or less per supercell over the range explored by the fluctuations (Table S8), far below the thermal energy at the transition, and a configuration displaced as far as δ=0.31 relaxes back to C2. The lattice can therefore fluctuate freely into P1 arrangements around the chiral minimum, while leaving the higher barrier separating the two handednesses (θ) untouched. The P1 structure is thus not a competing phase but the symmetry of instantaneous structures visited by the fluctuating chiral lattice. Quantizing δ as a single coordinate in this potential (Section S4) yields a ground state that is exactly symmetric, ⟨δ⟩=0, yet spreads broadly across the soft well through zero‐point motion alone.

This picture reconciles the seemingly contradictory symmetries reported in experiments. The static C2 lattice has already lost inversion and three‐fold rotational symmetries, which qualitatively accounts for the principal spectroscopic signatures, such as the splitting of degenerate optical phonon modes and their infrared activation. The fluctuations additionally remove the remaining twofold axis; because the spectroscopic fingerprints of this distortion grow with |δ| (mode splittings) or $\delta^{2}$ (mode intensities), they persist in the measured averages even though the average distortion vanishes, and vibrational probes can therefore detect no remaining point symmetry. Probes that instead average the atomic positions directly—diffraction within a uniform chiral domain, and scanning tunneling microscopy, whose topographic contrast is insensitive to δ (Figure 4a)—recover the chiral C2 structure.

**FIGURE 4:**
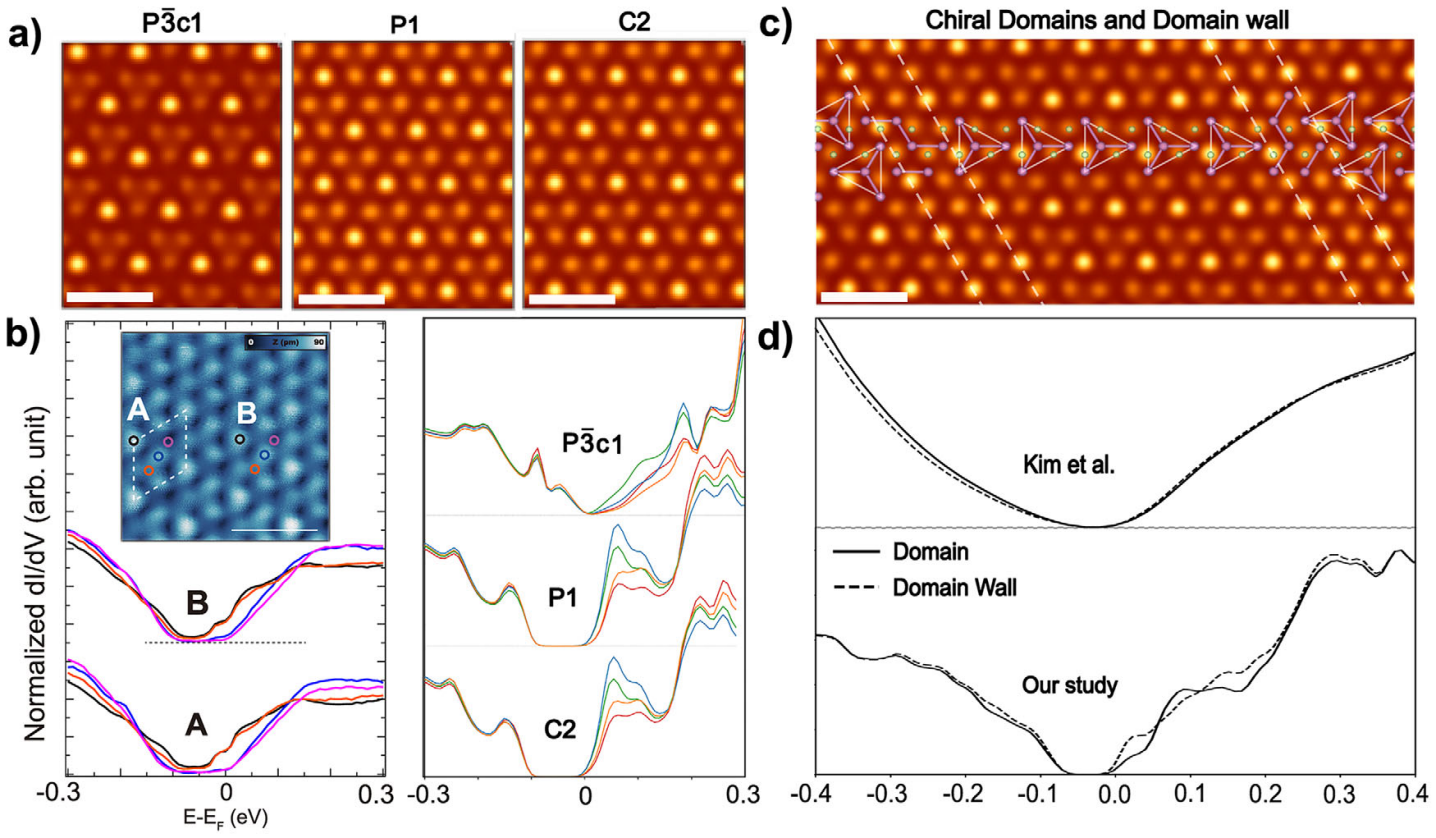
Simulated and experimental STM/STS. (a) Simulated constant‐current STM topographies (+120 meV). The achiral $P\bar{3}c1$ phase (left) exhibits a hexagonal superlattice, whereas the chiral C2 and P1 phases display nearly indistinguishable triangular motifs. This indistinguishable STM profile reflects the subtle intra‐trimer bond disproportionation of the nematic fluctuation within the C2 framework. (b) Experimental (left) and calculated (right) local electronic structure. Left: STM image (−0.2 V, 200 pA, 2.4×2.4 nm^2^) with spatially resolved dI/dV spectra (positions A and B). For dI/dV spectra measured on location B, vertical offsets are given for comparison (dashed line). Right: LDOS for the three candidate phases. The chiral C2 and P1 phases open a hard gap (∼80–100 meV), while $P\bar{3}c1$ remains pseudogapped near $E_{F}$. (c) Simulated topography with atomic overlay of a seamless domain wall connecting antipodal chiral C2 domains (white dashed lines). The half‐unit‐cell translational shift preserves trimerization and stacking topology across the boundary. (d) LDOS across the domain wall. Top: Experimental STS data adapted from Ref. [20] showing preserved gap magnitude in the domain (solid) and at the wall (dashed). Bottom: Calculated LDOS for the C2 wall, reproducing the absence of mid‐gap states despite local 1Q symmetry.

Having established this lattice‐driven C2 ground state, we can address two apparent tensions with prior literature. First, recent models propose that chirality originates from “charge‐lattice frustration,” invoking an independent electronic charge modulation, $\delta \rho_{L}$, that is symmetry‐mismatched with the lattice [41]. Standard DFT structural relaxations, however, couple the electronic density and ionic positions self‐consistently, not allowing an electronic order parameter that is variationally free from the lattice. Within this self‐consistent framework, we examined whether any lattice proxy for the proposed electronic modulation appears in the relaxed structure. Decomposing the C2 ground state into its symmetry components reveals that the Se‐only displacement associated with this mode is nearly two orders of magnitude smaller than the primary Ti‐centered lattice distortions (Table S9). This indicates that, within the standard adiabatic framework, the $2Q_{L}+1Q_{M}$ mechanism provides a sufficient lattice‐driven origin without requiring an additional frustration term. Second, inelastic X‐ray scattering shows that the M‐point phonon remains hard at $T_{c}$ [41, 43, 44]. This observation is not incompatible with the C2 structure, because the relaxation proceeds dynamically within the single phase transition. The dominant L‐mode condensation initially forms a $P\bar{3}c1$ configuration that is itself unstable to transverse shear distortions. The lattice slides toward the C2 global minimum along this shear coordinate without requiring the M phonon to independently soften or to drive a separate thermodynamic transition. The participation of the M‐point phonon in the transition through the shear mode is fully consistent with the previous experimental works [41, 43].

### 2.2 Comparison with Experimental Observations

Although total energy calculations show that the chiral C2 structure is stable, this model must also reproduce the experimental electronic and spatial signatures without relying on independent electronic orders. Previous work established that the C2 structure excellently reproduces the chiral topography of STM images [20]. Here, we also simulated the STM topography for the triclinic P1 configurations. Since the P1 symmetry represents a continuous path along this soft manifold rather than a single fixed minimum, we selected a representative configuration (δ≈0.3, Table S6). As shown in Figure 4a, this highly asymmetric P1 configuration reproduces the chiral STM topography just as well as the C2 ground state. The nearly indistinguishable topographies show that the STM contrast is generated by the structural motif itself and is insensitive to δ: as a surface‐sensitive probe, STM images the chiral order irrespective of the instantaneous configuration of the fluctuating lattice, whereas vibrational spectroscopies respond to the instantaneous local bonding environment. The two apparently exclusive symmetry assignments therefore describe different averages of the same fluctuating lattice, rather than two different phases.

We next examine the single‐particle electronic structure of the competing phases. The achiral $P\bar{3}c1$ phase exhibits a semimetallic local density of states (LDOS), which is inconsistent with our spatially resolved dI/dV spectra (Figure 4b) and previous measurements [20]. In sharp contrast, the chiral C2 distortion spontaneously generates an insulating surface gap (∼100 meV). Previous theoretical models required empirical electronic correlation corrections to reproduce this gap magnitude [20]. Our calculations demonstrate that a hard gap emerges from first‐principles without such ad hoc parameters. This indicates that the lattice symmetry breaking provides the primary structural driving force for the insulating state, even if correlation effects are ultimately required for a quantitative match (details on functional benchmarking are provided in the Supporting Information). Consistent with this picture, self‐consistent many‐body calculations including excitonic correlations find an exciton binding energy an order of magnitude smaller than the gap, and show that insulating spectra arise from lattice distortions, static or fluctuating [35]; likewise, the pseudogap and replica‐band signatures of the normal phase are reproduced by dynamical electron‐phonon self‐energies without excitonic terms [34]. Furthermore, the calculated bulk band structure yields a smaller indirect gap (∼20–30 meV, Figure S4), which is consistent with bulk angle‐resolved photoemission spectroscopy measurements [21, 45]. Band dispersions sampled along the nematic coordinate (δ≲0.3) preserve this insulating topology, indicating that the dynamic structural fluctuations do not alter the fundamental electronic character.

The behavior of the C2 ground state can be further examined at topological defects. Figure 4c illustrates a domain wall that separates connecting antipodal (opposite‐handed) chiral domains. At this boundary, the Ti‐trimer network is locally disrupted, forming a $1Q_{L}$‐like linear structure to bridge the two domains. Despite this structural disruption, our calculated local density of states (LDOS) indicates that the energy gap remains open across the boundary (Figure 4d). This preserved gap is consistent with the experimental tunneling spectroscopy reported by Kim et al. [20], which shows an absence of mid‐gap states at the wall. This insulating nature distinguishes 1*T*‐TiSe_2_ from the domain walls with distinct in‐gap states as reported in conventional Peierls systems like 1*T*‐TaS_2_ [46]. Furthermore, our modeling suggests that this gapped boundary behavior remains stable in the presence of nematic fluctuations (δ≠0), as the intra‐trimer bond stretching along the soft manifold does not alter the underlying geometric constraints of the boundary orientations.

## 3 Discussion

These findings suggest that the microscopic origin of the insulating state in 1*T*‐TiSe_2_ is consistent with a lattice‐driven mechanism. By identifying the chiral C2 phase as the global minimum within a soft nematic manifold, we demonstrate that cooperative condensation of primary L‐ and M‐point phonon modes generates sufficient band reconstruction to open an insulating gap consistent with experiments. While quantitative accuracy in the gap magnitude requires the meta‐GGA $r^{2}\text{SCAN}+r\text{VV10}$ functional, the qualitative trend is robust across different approximations. Specifically, the chiral C2 phase consistently opens a hard gap while the achiral $P\bar{3}c1$ phase remains metallic in standard semilocal GGAs (PBE+D3, optB86b; Figure S1). This functional independence indicates that the existence of the CDW gap is primarily controlled by the chiral C2 symmetry breaking, while nonlocal exchange and excitonic correlations likely control its precise magnitude and fine structure [29, 30, 31].

The soft nematic energy surface unifies the divergent experimental pictures of the TiSe_2_ ground state. The flat potential along the nematic coordinate δ allows the dynamical fluctuation into the triclinic P1 configuration at a sub‐meV energetic cost. A related mechanism operates in the monolayer limit, where long‐wavelength acoustic‐phonon fluctuations on an asymmetric potential surface generate unequal amplitudes of the three charge‐density‐wave components, stabilizing a chiral average structure at low temperature, while relaxing the fluctuation‐averaged structure recovers the symmetric achiral form [47]. In the bulk phase studied here, the directional $2Q_{L}+1Q_{M}$ mixing is instead selected statically at T=0 by the quartic mode coupling, and the fluctuations subsequently explore the soft δ direction around the chiral minimum. The two mechanisms are complementary: both act on near‐flat, asymmetric potential energy surfaces, and both yield average structures whose symmetry is higher than that of the instantaneous configurations. While the chiral C2 phase is established as the definitive T=0 ground state, the shallow energy landscape and sub‐meV nematic fluctuations suggest that finite‐temperature properties may be sensitive to lattice anharmonicity. The temperature‐dependent phonon renormalization, including the evolution of the M‐mode response, falls outside the scope of the present static zero‐temperature framework. Ab initio molecular dynamics or finite‐temperature lattice dynamics would be valuable extensions to explore whether anharmonic stabilization effects contribute to these experimental signatures.

The small energy difference between the chiral C2 global minimum and the achiral $P\bar{3}c1$ saddle point (ΔE≈1.6 meV/supercell) in the present free energy surface can consistently interpret very recent small‐area‐electron‐diffraction measurements [48]. This work reported bulk phase separation into regions with and without M‐point diffraction peaks. Because the energetic cost to flip the chirality is so small, the pristine bulk is highly susceptible to nanoscale domain fragmentation upon cooling. If the bulk forms a dense mosaic of static C2 domains that are smaller than the electron beam's transverse coherence length, the out‐of‐phase $+1Q_{M}$ and $-1Q_{M}$ components would destructively interfere, effectively extinguishing the elastic M‐point diffraction peaks. Conversely, local pinning due to defects or strain gradients would stabilize macroscopic C2 domains larger than the coherence length, allowing the M‐peaks to emerge. Within a uniform chiral domain, coherently diffracting probes average to the C2‐centered structure; the achiral average structure resolved by early crystallographic analyses then follows naturally if the sample contains comparable populations of left‐ and right‐handed domains, which is also a statistical picture independently proposed on theoretical grounds [40]. This may explain why surface‐sensitive probes like STM capture a chiral configuration, possibly unifying apparently contradictory surface and bulk measurements.

## 4 Summary

Chiral charge‐density waves in 1*T*‐TiSe_2_ can be understood as a lattice‐driven instability in which cooperative condensation of L‐ and M‐point phonon modes stabilizes a monoclinic C2 ground state and opens an insulating gap consistent with STM and ARPES experiments. This C2 phase sits at the center of a soft nematic energy manifold, a single shallow valley whose quantum ground state is exactly centered (⟨δ⟩=0) yet broad enough to visit fully asymmetric P1 arrangements even at zero temperature. A chiral lattice fluctuating over this shallow well reconciles the two experimental pictures that have long appeared contradictory: structure probes (such as STM topography and diffraction within a uniform chiral domain) recover the average C2 lattice, while vibrational spectroscopies measure the instantaneous local symmetry and find none remaining. More broadly, this mechanism shows how chiral‐nematic CDWs can emerge from the interplay of chiral lattice instabilities and nematic fluctuations, providing a concrete structural framework for interpreting symmetry breaking in TiSe_2_ and related quantum materials.

## 5 Methods

### 5.1 Computational Details

Density functional theory calculations were performed using the projector augmented‐wave (PAW) method [49] as implemented in the vasp code [50, 51]. Exchange‐correlation interactions were treated using the *r* ^2^SCAN+rVV10 functional [52, 53, 54] with a 520 eV plane‐wave cutoff. We used Γ‐centered k‐point meshes of 12×12×6 for the primitive cell, with correspondingly reduced grids for the 2×2×2 supercells. Structural optimizations were performed with convergence criteria of $10^{-8}$ eV for total energy and $10^{-3}$ eV/Å for atomic forces. Spin–orbit coupling was included in electronic structure calculations for accurate determination of bandgaps and dispersions, while structural energetics were found to be negligibly affected by relativistic effects. To test the lattice instability mechanism, we generated initial atomic distortions corresponding to the L‐point ($\Gamma_{7}^{3}$) and M‐point ($\Gamma_{5}^{3}$) irreducible representations identified in Kim et al., [41] following the instability analysis by Subedi [37]. Symmetry identification was performed using the pymatgen library [55]. Scanning tunneling microscopy images were simulated within the Tersoff‐Hamann approximation [56]. Data were processed using vaspkit [57].

### 5.2 Experimental Details

Atomically resolved imaging and spectroscopy were performed under ultrahigh vacuum (UHV) at 2.2 K using a commercial low‐temperature scanning tunneling microscope (STM) and Pt‐Ir tips. For scanning tunneling spectroscopy (STS) measurements, the dI/dV spectra were recorded with the lock‐in technique using a 15 mV modulation voltage. Clean 1*T*‐TiSe_2_ surface was prepared by UHV cleavage at room temperature.

## Author Contributions

H.W.Y. conceived the project, directed the overall narrative of the research, and edited and finalized the manuscript. A.A.A. performed the ab initio calculations, derived the theoretical model, wrote the original draft of the manuscript, and prepared the figures. E.D. conducted the STM/STS experiments and provided all experimental data. J.W.P. provided project supervision and scientific guidance, and reviewed the manuscript.

## Conflicts of Interest

The authors declare no conflicts of interest.

## Supporting information


**Supporting File 1**: advs78243‐sup‐0001‐SuppMat.pdf.

**Supporting File 2**: advs78243‐sup‐0002‐SuppMat.zip.

## Data Availability

The data that support the findings of this study are available from the corresponding author upon reasonable request.
