# Supplementary material for: Chiral‐Nematic Lattice Instability in Charge Density Wave of 1T‐TiSe2
Source: Adv Sci (Weinh). 2026 Oct 20:e78243. Online ahead of print. doi: 10.1002/advs.78243 (PMC13683907; doi:10.1002/advs.78243)
Supplement: Supplementary file 1 [file ADVS-9999-e78243-s002.pdf]

Supporting Information

Chiral-Nematic Lattice Instability in Charge Density Wave of $1T$-$TiSe_2$

Abdurrafi Ahsan Alghifari, Euihwan Do, Jae Whan Park, and Han Woong Yeom*

*E-mail: yeom@postech.ac.kr

Contents

S1 Functional Independence of the Structural Gap **2**

S2 Ground-State and Metastable Crystal Structures **4**
S2.1 Quantitative Structural Metrics 8

S3 Landau Free Energy Expansion **10**
S3.1 Symmetry-Adapted Functional Form 10
S3.2 Linear Regression of DFT Energetics 10
S3.3 Effective Free Energy Surface 11

S4 Quantum fluctuation along the nematic collective coordinate **13**
S4.1 Construction of the potential and the effective mass 13
S4.2 Ground state of the one-dimensional coordinate 13
S4.3 Limitations 13

S5 Band Unfolding and ARPES Simulation **15**

S1. Functional Independence of the Structural Gap

Isolating the structural contribution to the insulating gap requires demonstrating that the gap opens in the chiral phase even in the absence of non-local exchange or empirical correlations. Figure S1 compares the electronic density of states (DOS) for the achiral $P\bar{3}c1$ (orange) and chiral $C2$ (blue) phases across three functionals: semilocal PBE+D3 [1,2] and optB86b-vdW [3], and the meta-GGA r^2SCAN+rVV10 [4,5].

For standard GGAs (PBE+D3, optB86b-vdW), the $P\bar{3}c1$ phase exhibits finite spectral weight at the Fermi level (E_F), consistent with metallic character, whereas the $C2$ phase opens a distinct gap of approximately 30 meV. The r^2SCAN+rVV10 functional predicts a semimetallic $P\bar{3}c1$ phase with suppressed DOS at E_F, while opening a larger hard gap of ∼80–100 meV in the $C2$ phase. The emergence of a gap exclusively in the chiral phase across all functionals—despite their inability to capture excitonic correlations—confirms that the lattice symmetry breaking alone is sufficient to drive the insulating transition.

Table S1 provides the corresponding lattice parameter benchmarks.

Table S1. Comparison of Lattice parameters and c-axis errors for the $TiSe_2$ normal phase. Data for the TASK+D2, and MVS+D2 functionals are taken from Yin *et al.*, [6] while HSE06 values are from Hellgren *et al.*. [7]

Functional	a (Å)	c (Å)	c-error (%)
Experiment	3.540	6.007	—
PBE+D3	3.512	5.946	-1.0
r^2SCAN+rVV10	3.539	6.033	0.4
TASK+D2	3.659	7.456	24.2
MVS+D2	3.607	9.891	64.8
HSE06	3.540	6.104	1.6

A parallel functional-independence test can be carried out for the structural energetics. All competing phases of Table 1 of the main text were fully relaxed with the semilocal PBE(+D3) functional [1,2], using otherwise identical computational settings (Table S2). Although the condensation energies are compressed to about two-thirds of the r^2SCAN+rVV10 values, the key hierarchy is preserved: at equal propagation-vector count the L-mode condensates remain more strongly bound than the M-mode ones, and the chiral $C2$ phase remains the global minimum, lying ∼0.5 meV below $P\bar{3}c1$ per supercell (versus 1.6 meV with r^2SCAN+rVV10).

Table S2. Phase energetics for two exchange–correlation functionals, relative to the pristine $P\bar{3}m1$ structure (meV per $2 \times 2 \times 2$ cell). The r^2SCAN+rVV10 column reproduces Table 1 of the main manuscript for direct comparison.

Family	Distortion	Symmetry	ΔE (r^2SCAN+rVV10)	ΔE (PBE+D3)
Reference	—	$P\bar{3}m1$	0.00	0.00
Pure M	$1Q_M$	$P2/c$	−9.7	−7.8
	$2Q_M$	$C2/m$	−13.4	−8.1
	$3Q_M$	$P321$	−15.5	−8.3
Pure L	$1Q_L$	$C2/c$	−10.7	−8.7
	$2Q_L$	$C2/m$	−14.9	−10.7
	$3Q_L$	$P\bar{3}c1$	−17.2	−12.0
Mixed	$1Q_M + 1Q_L$	$P\bar{1}$	−14.5	−8.4
	$2Q_M + 1Q_L$	$C2/c$	−17.7	−10.7
	$2Q_L + 1Q_M$	$C2$	−18.8	**−12.5**

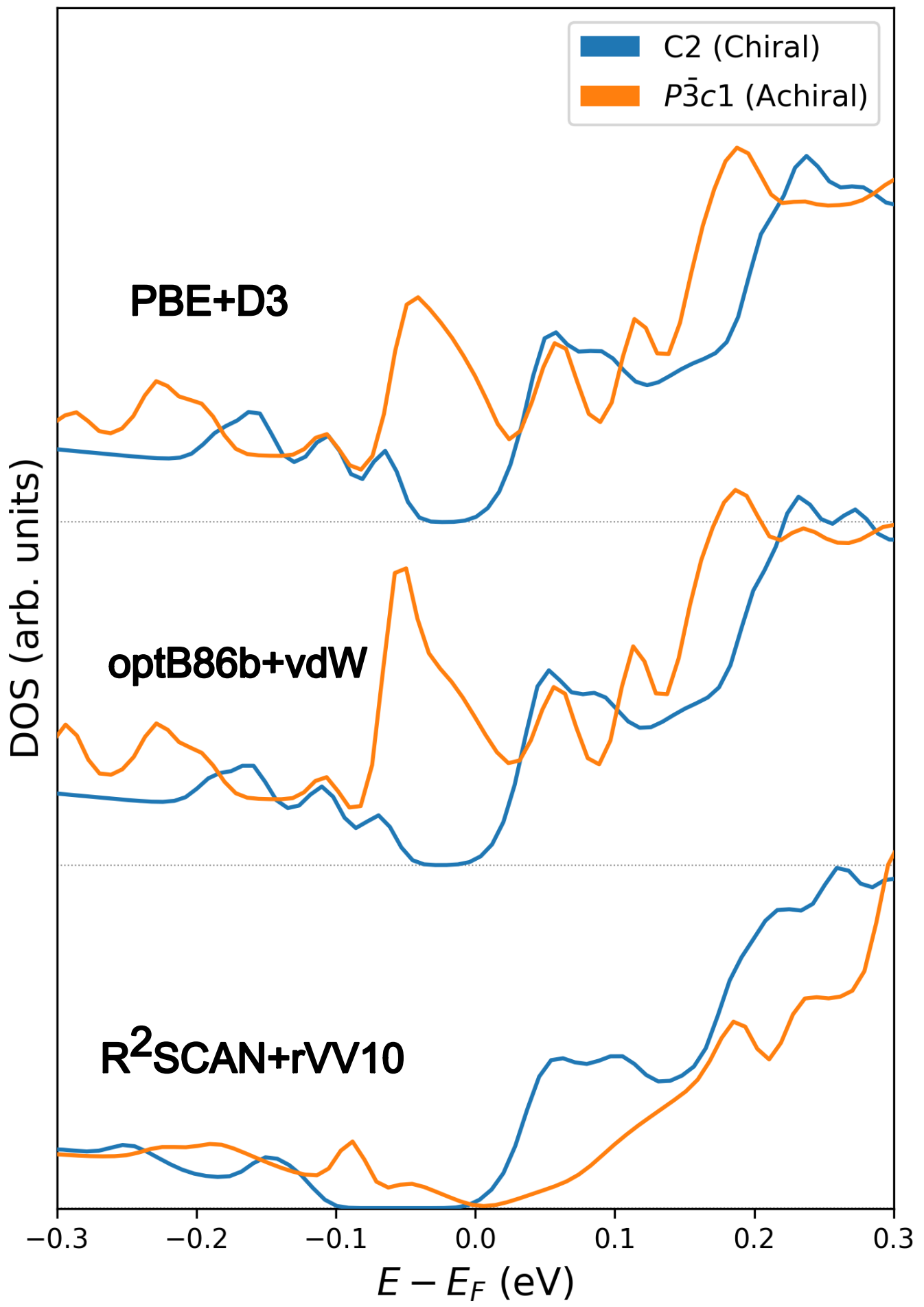

Figure S1. Integrated Density of States for different functionals. PBE+D3 and optB86b+vdW show a similar curve to each other but different to r^2SCAN+rVV10. Despite being metallic in achiral phase ($P\bar{3}c1$) compared to semimetallic in r^2SCAN+rVV10, all tested functionals show a hard gap opening in the chiral phases ($C2$ and $P1$), with ~30 meV and ~80–100 meV for GGA and meta-GGA, respectively. The selected GGA functionals also exhibit better qualitative agreement with meta-GGA in the chiral phases compared to the achiral one.

S2. Ground-State and Metastable Crystal Structures

We provide fully relaxed structural parameters for the phases analyzed in the energetic hierarchy. All coordinates are given in fractional units of the lattice vectors. Wyckoff positions represent the approximate site symmetry corresponding to the ideal high-symmetry prototype; coordinates deviate slightly from ideal positions due to numerical convergence and the relaxed distortion amplitudes.

Table S3. Structural parameters for the parent phase $P\bar{3}m1$ (#164). Lattice constants: $a = b = 7.07944$ Å, $c = 12.06332$ Å. Wyckoff positions represent the approximate site symmetry. Coordinates are fully relaxed and deviate from ideal positions due to the reconstruction in the 2×2×2 supercell.

Atom	Wyckoff	x	y	z
Ti(1)	1*a*	0.00000	0.00000	0.25000
Ti(2)	2*d*	0.50000	0.00000	0.25000
Ti(3)	2*d*	0.00000	0.50000	0.25000
Ti(4)	2*d*	0.50000	0.50000	0.25000
Ti(5)	1*b*	0.00000	0.00000	0.75000
Ti(6)	2*d*	0.50000	0.00000	0.75000
Ti(7)	2*d*	0.00000	0.50000	0.75000
Ti(8)	2*d*	0.50000	0.50000	0.75000
Se(1)	2*d*	0.16667	0.33333	0.37606
Se(2)	2*d*	0.66667	0.33333	0.37606
Se(3)	2*d*	0.16667	0.83333	0.37606
Se(4)	2*d*	0.66667	0.83333	0.37606
Se(5)	2*d*	0.33333	0.16667	0.12394
Se(6)	2*d*	0.83333	0.16667	0.12394
Se(7)	2*d*	0.33333	0.66667	0.12394
Se(8)	2*d*	0.83333	0.66667	0.12394
Se(9)	2*d*	0.16667	0.33333	0.87606
Se(10)	2*d*	0.66667	0.33333	0.87606
Se(11)	2*d*	0.16667	0.83333	0.87606
Se(12)	2*d*	0.66667	0.83333	0.87606
Se(13)	2*d*	0.33333	0.16667	0.62394
Se(14)	2*d*	0.83333	0.16667	0.62394
Se(15)	2*d*	0.33333	0.66667	0.62394
Se(16)	2*d*	0.83333	0.66667	0.62394

Table S4. Structural parameters for the canonical phase $P\bar{3}c1$ (#165). Lattice constants: $a = b = 7.07445$ Å, $c = 12.10094$ Å.

Atom	Wyckoff	x	y	z
Ti(1)	2a	0.00000	0.00860	0.25000
Ti(2)	6f	0.49140	0.99140	0.25000
Ti(3)	2b	0.00000	0.50000	0.25000
Ti(4)	6f	0.50860	0.50000	0.25000
Ti(5)	2a	0.00000	0.99140	0.75000
Ti(6)	6f	0.50860	0.00860	0.75000
Ti(7)	2b	0.00000	0.50000	0.75000
Ti(8)	6f	0.49140	0.50000	0.75000
Se(1)	4d	0.16372	0.33064	0.37615
Se(2)	12g	0.66692	0.33628	0.37615
Se(3)	12g	0.16936	0.83308	0.37615
Se(4)	4d	0.66667	0.83333	0.37560
Se(5)	4d	0.33333	0.16667	0.12440
Se(6)	12g	0.83628	0.16692	0.12385
Se(7)	12g	0.33308	0.66936	0.12385
Se(8)	12g	0.83064	0.66372	0.12385
Se(9)	12g	0.16936	0.33628	0.87615
Se(10)	12g	0.66692	0.33064	0.87615
Se(11)	12g	0.16372	0.83308	0.87615
Se(12)	4d	0.66667	0.83333	0.87560
Se(13)	4d	0.33333	0.16667	0.62440
Se(14)	12g	0.83064	0.16692	0.62385
Se(15)	12g	0.33308	0.66372	0.62385
Se(16)	12g	0.83628	0.66936	0.62385

Table S5. Structural parameters for the chiral ground state $C2$ (#5). Lattice constants: $a = 7.07553$ Å, $b = 7.07553$ Å, $c = 12.10981$ Å, $\alpha = 90.00°$, $\beta = 90.01°$, $\gamma = 120.01°$.

Atom	Wyckoff	x	y	z
Ti(1)	4c	0.00000	0.00860	0.25000
Ti(2)	4c	0.49107	0.99137	0.25002
Ti(3)	4c	0.00000	0.49975	0.25000
Ti(4)	4c	0.50893	0.50029	0.24998
Ti(5)	4c	0.00000	0.99975	0.75000
Ti(6)	4c	0.50893	0.00029	0.74998
Ti(7)	4c	0.00000	0.50860	0.75000
Ti(8)	4c	0.49107	0.49137	0.75002
Se(1)	4c	0.16352	0.33066	0.37605
Se(2)	4c	0.66693	0.33628	0.37603
Se(3)	4c	0.16945	0.83314	0.37600
Se(4)	4c	0.66664	0.83319	0.37554
Se(5)	4c	0.33336	0.16655	0.12446
Se(6)	4c	0.83648	0.16714	0.12395
Se(7)	4c	0.33307	0.66934	0.12397
Se(8)	4c	0.83055	0.66369	0.12400
Se(9)	4c	0.16945	0.33314	0.87600
Se(10)	4c	0.66664	0.33319	0.87554
Se(11)	4c	0.16352	0.83066	0.87605
Se(12)	4c	0.66693	0.83628	0.87603
Se(13)	4c	0.33307	0.16934	0.62397
Se(14)	4c	0.83055	0.16369	0.62400
Se(15)	4c	0.33336	0.66655	0.62446
Se(16)	4c	0.83648	0.66714	0.62395

Table S6. Structural parameters for the extreme nematic limit $P1$ (#1) corresponding to $\delta = 0.31$. Lattice constants: $a = 7.07557$ Å, $b = 7.07557$ Å, $c = 12.10977$ Å, $\alpha = 90.01°$, $\beta = 89.99°$, $\gamma = 119.99°$.

Atom	Wyckoff	x	y	z
Ti(1)	1a	0.99776	0.00692	0.25001
Ti(2)	1a	0.49284	0.99308	0.25001
Ti(3)	1a	0.00220	0.50198	0.25000
Ti(4)	1a	0.50720	0.49802	0.24998
Ti(5)	1a	0.00220	0.00198	0.75000
Ti(6)	1a	0.50720	0.99802	0.74998
Ti(7)	1a	0.99776	0.50692	0.75001
Ti(8)	1a	0.49284	0.49308	0.75001
Se(1)	1a	0.16419	0.33120	0.37590
Se(2)	1a	0.66614	0.33558	0.37602
Se(3)	1a	0.16903	0.83243	0.37603
Se(4)	1a	0.66732	0.83392	0.37562
Se(5)	1a	0.33408	0.16725	0.12438
Se(6)	1a	0.83576	0.16632	0.12397
Se(7)	1a	0.33236	0.66895	0.12395
Se(8)	1a	0.83112	0.66435	0.12413
Se(9)	1a	0.16903	0.33243	0.87603
Se(10)	1a	0.66732	0.33392	0.87562
Se(11)	1a	0.16419	0.83120	0.87590
Se(12)	1a	0.66614	0.83558	0.87602
Se(13)	1a	0.33236	0.16895	0.62395
Se(14)	1a	0.83112	0.16435	0.62413
Se(15)	1a	0.33408	0.66725	0.62438
Se(16)	1a	0.83576	0.66632	0.62397

S2.1. Quantitative Structural Metrics

Table S7 summarizes the bond length distribution within the Ti-sublattice for the analyzed phases. The parent phase $P\bar{3}m1$ exhibits uniform Ti-Ti distances (3.540 Å) corresponding to the high-symmetry hexagonal lattice. The distorted phases exhibit significant bond contraction. The $P\bar{3}c1$ and $C2$ phases show uniform short bonds centered at 3.476 Å, representing a $\sim$1.8% contraction relative to the parent phase that drives Ti-trimer formation. The nematic $P1$ configuration exhibits bond length disproportionation within the trimer unit ($\Delta d \approx 0.018$ Å), confirming the structural signature of the soft nematic manifold explored in the main text.

Table S7. Ti-sublattice nearest-neighbor bond length distribution ($d_{\text{Ti-Ti}}$). The parent phase distance is 3.540 Å. Δd indicates the spread of bonds within the trimer unit.

Phase	Space Group	$d_{\text{Ti-Ti}}$ Range (Å)	Δd (Å)	Contraction (%)
Parent ($P\bar{3}m1$)	#164	3.540 (Uniform)	0.000	0.0
Canonical ($P\bar{3}c1$)	#165	3.476 (Uniform)	0.000	-1.8
Chiral ($C2$)	#5	3.477 (Uniform)	0.000	-1.8
Nematic ($P1$)	#1	3.468 – 3.486	0.018	-1.8

Table S8. Energy cost and structural forces along the nematic distortion coordinate. The dimensionless nematic parameter $\delta = (d_{L,2} - d_{L,3})/(d_{L,2} + d_{L,3})$ quantifies the amplitude imbalance between the active transverse L-modes. This theoretical coordinate is mapped to physical dimensions via the intra-trimer bond disproportionation (Δd) and the maximum absolute atomic displacement (ΔR_{max}) relative to the $C2$ ground state. The maximum force (F_{max}) indicates the magnitude of atomic forces prior to tight relaxation; all configurations in this range relax back to the $C2$ minimum without encountering any other minimum.

Nematic Parameter δ	Bond Disproportionation Δd (mÅ)	Max Atomic Disp. ΔR_{max} (mÅ)	Energy Cost ΔE (meV)	Maximum Force F_{max} (eV/Å)
0.3100	18.1	17.7	1.69	0.020
0.2625	12.5	15.6	1.04	0.018
0.1897	10.1	12.4	0.50	0.011
0.1558	8.8	10.7	0.33	0.010
0.1221	7.2	8.8	0.21	0.009
0.0861	5.4	6.6	0.12	0.007
0.0666	4.3	5.9	0.09	0.006
0.0457	3.2	4.6	0.05	0.005
0.0150	2.4	2.8	0.02	0.004
0.0000	1.8[a]	0.0	0.00	0.000

[a]The residual 1.8 mÅ disproportionation in the nominal ground state reflects the finite force-convergence tolerance (10^{-3} eV/Å) of the DFT structural relaxation, consistent with the slight L-mode asymmetry noted in Table S9.

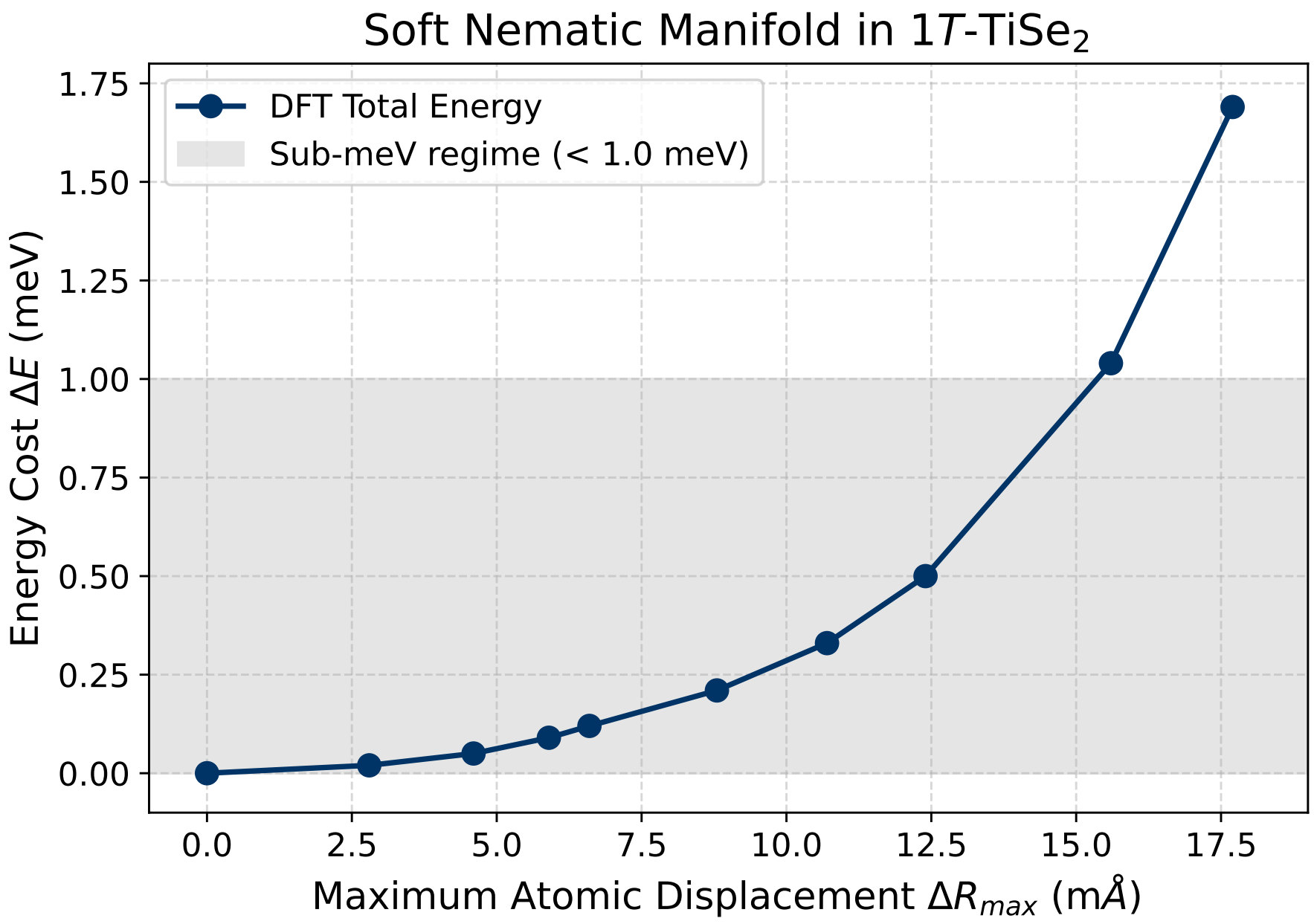

Figure S2. Energy landscape of the soft nematic manifold. Density functional theory calculations of the total energy cost (ΔE) as a function of the maximum atomic displacement (ΔR_{max}) relative to the chiral $C2$ ground state. The distortion path describes the continuous transition from the $C2$ minimum into triclinic $P1$ configurations via intra-trimer bond disproportionation. The landscape exhibits an exceptionally shallow harmonic well. The shaded gray area highlights the sub-meV fluctuation regime (< 1.0 meV). This broad, near-zero-energy basin allows the lattice to dynamically sample fully asymmetric configurations, reconciling the time-averaged $C2$ chirality with instantaneous $P1$ symmetry loss.

Table S9. Symmetry-mode projection amplitudes for fully relaxed CDW structures. Amplitudes for the atomic displacement modes along the three propagation vectors ($\mathbf{q}_1, \mathbf{q}_2, \mathbf{q}_3$), extracted from fully relaxed DFT coordinates. Values are presented in milli-Ångströms (mÅ), converted from fractional units using the nominal in-plane lattice parameter $a = 7.075$ Å.

Phase	Space Group	Γ_7^3 (L-mode, Ti)	Γ_5^3 (M-mode, Ti)	Γ_4^3 (Charge, Se)
Achiral ($3Q_L$)	$P\bar{3}c1$ (#165)	$(30.4, -30.4, -30.4)$	$(0.0, 0.0, 0.0)$	$(0.0, 0.0, 0.0)$
Chiral ($2Q_L + 1Q_M$)	$C2$ (#5)	$(0.0, -31.5, -31.6)$[a]	$(29.5, 0.0, 0.0)$	$(0.0, 0.6, 0.6)$

[a]The slight difference between the secondary axes (31.5 vs 31.6 mÅ) reflects the finite force-convergence tolerance (10^{-3} eV/Å) of the DFT structural relaxation; an ideal $C2$ symmetry dictates identical amplitudes.

S3. Landau Free Energy Expansion

S3.1. Symmetry-Adapted Functional Form

Following the symmetry analysis of the $P\bar{3}m1$ parent phase,[8] we construct a Landau free energy expansion in terms of the primary L-point (Γ_7^3) and M-point (Γ_5^3) phonon order parameters, denoted $\mathbf{d}_L$ and $\mathbf{d}_M$, respectively. The functional is restricted to lowest-order invariants permitted by momentum conservation and the D_{3d} point group.

The total free energy is decomposed into single-mode sectors and an interaction term:

$$F(\mathbf{d}_L, \mathbf{d}_M) = F_L(\mathbf{d}_L) + F_M(\mathbf{d}_M) + F_{int}(\mathbf{d}_L, \mathbf{d}_M). \tag{S1}$$

The single-mode sectors encapsulate the harmonic drive, anharmonic stabilization, and intra-component repulsion:

$$F_L = a_L \sum_i d_{Li}^2 + b_L \sum_i d_{Li}^4 + \lambda_L \sum_{i<j} d_{Li}^2 d_{Lj}^2, \tag{S2}$$

$$F_M = a_M \sum_i d_{Mi}^2 + b_M \sum_i d_{Mi}^4 + \lambda_M \sum_{i<j} d_{Mi}^2 d_{Mj}^2, \tag{S3}$$

where $i = 1,2,3$ indexes the three propagation vectors constituting the star of L and M. A negative quadratic coefficient ($a < 0$) indicates a harmonic instability, while a positive quartic coefficient ($b > 0$) ensures saturation. Crucially, positive λ represents the repulsive interaction between different propagation vectors of the same branch, penalizing simultaneous condensation (e.g., the $3Q$ state relative to $1Q$ states).

The interaction sector allows two distinct fourth-order invariants coupling the L and M amplitudes:

$$F_{int} = \kappa \sum_i (d_{Li}^2 d_{Mi}^2) + \gamma \sum_{i \neq j} (d_{Li}^2 d_{Mj}^2). \tag{S4}$$

Here, κ governs the ”on-site” energetic penalty between condensates sharing the same propagation vector, while γ governs the ”cross-site” coupling between different propagation vectors. Note that trilinear couplings of the form d_L^3 or $d_L^2 d_M$ are forbidden by the momentum conservation laws associated with the $P\bar{3}m1$ Brillouin zone topology, leaving these quartic terms as the leading-order interaction.

S3.2. Linear Regression of DFT Energetics

To determine the phenomenological coefficients in Equations (S2–S4), we performed a least-squares linear regression against the DFT total energies of the structural phases listed in Table 1 (main text) and their neighboring distortion pathways. The amplitudes $\mathbf{d}_L$ and $\mathbf{d}_M$ were extracted directly from the atomic displacement vectors relative to the $P\bar{3}m1$ parent phase. The regression yields an R^2 coefficient of determination of 0.994, confirming that the inclusion of intra-component coupling terms (λ) is necessary to capture the potential energy landscape quantitatively.

For the extraction of phenomenological coefficients, the dimensionless fractional mode amplitudes were mapped to Cartesian physical displacements (Å) using the nominal in-plane lattice parameter $a = 7.075$ Å. The minor secondary strains associated with the fully relaxed unit cells ($\Delta a/a < 0.1\%$) are implicitly integrated into the effective quartic coefficients.

The extracted coefficients are:

$$\begin{aligned} &a_L = -1.18 \times 10^4 \text{ meV} \cdot \text{Å}^{-2}, \quad && b_L = 3.24 \times 10^6 \text{ meV} \cdot \text{Å}^{-4}, \quad && \lambda_L = 1.38 \times 10^6 \text{ meV} \cdot \text{Å}^{-4}, \\ &a_M = -1.00 \times 10^4 \text{ meV} \cdot \text{Å}^{-2}, && b_M = 2.58 \times 10^6 \text{ meV} \cdot \text{Å}^{-4}, && \lambda_M = 1.14 \times 10^6 \text{ meV} \cdot \text{Å}^{-4}, \\ &\kappa = 1.82 \times 10^7 \text{ meV} \cdot \text{Å}^{-4}, && \gamma = 1.46 \times 10^6 \text{ meV} \cdot \text{Å}^{-4}. \end{aligned} \tag{S5}$$

Here, a and b denote the quadratic (harmonic) and quartic (anharmonic) coefficients, while λ, κ, and γ characterize the various fourth-order interactions.

The negative quadratic coefficients ($a_L, a_M < 0$) confirm the dual-phonon instability, while the positive quartic coefficients ($b_L, b_M > 0$) ensure saturation of the condensate amplitude. The intra-component couplings

($\lambda_L, \lambda_M > 0$) are comparable in magnitude to the single-mode stiffness terms (b). This repulsion renormalizes the effective stiffness in the $3Q$ limit, suppressing the amplitude of the condensate and preventing the energy of the $3Q$ phase from being a simple linear sum of its $1Q$ components.

The cross-branch interaction κ ($\sim 1.8 \times 10^7$ meV $\cdot$ Å^{-4}) is significantly larger than the single-mode stiffness ($b \sim 3.2 \times 10^6$ meV $\cdot$ Å^{-4}), indicating strong repulsion between L and M distortions sharing the same propagation axis. Physically, this reflects the incompatibility of the anti-phase trimerization (L) and uniform interlayer shear (M) displacements acting on the same crystallographic chain. To minimize the large penalty imposed by κ (which forbids $L+M$ mixing on single axes), the system partitions the order parameters onto distinct axes. This selective condensation stabilizes the $2Q_L + 1Q_M$ configuration, which avoids the penalties associated with simultaneous occupation of any single propagation vector by both modes.

S3.3. Effective Free Energy Surface

To visualize the topological structure of the ground state, we parameterize the free energy landscape using collective coordinates derived from the vector order parameters. We define:

- **Chiral Mixing Angle (θ):** Rotation of the order parameter on the primary propagation axis $\mathbf{q}_1$. This transforms the condensate on this axis from the L-mode to the M-mode:

$$d_{L1} = \eta_0 \cos\theta, \quad d_{M1} = \eta_0 \sin\theta. \tag{S6}$$

  The condensate amplitudes on the remaining two propagation axes (d_{L2}, d_{L3}) are held fixed at their L-mode values. Consequently, $\theta = 0$ corresponds to the canonical $P\bar{3}c1$ ($3Q_L$) configuration, while $\theta = \pi/2$ corresponds to the onset of C_2 chirality ($2Q_L + 1Q_M$).

- **Nematic Amplitude Imbalance (δ):** Relative amplitude difference between the secondary axes:

$$d_{L2} = \eta_0(1+\delta), \quad d_{L3} = \eta_0(1-\delta). \tag{S7}$$

  This coordinate quantifies the bond disproportionation degree of rotational symmetry breaking orthogonal to the primary chiral axis.

Substituting these coordinate constraints into the full Landau expansion (Equations S1–S4) yields the effective 2D energy surface. Note that because this specific pathway involves M-mode condensation on only one axis ($i = 1$), the intra-component repulsion λ_M vanishes identically ($d_{Mj} = 0$ for $j \neq 1$). However, the L-mode repulsion λ_L acts between all three axes. Collecting terms by their trigonometric and polynomial dependence on θ and δ, the energy surface takes the form:

$$F_{\text{eff}}(\theta, \delta) = C + B_\theta \sin^2\theta + A_\theta \sin^4\theta + B_\delta \delta^2 + A_\delta \delta^4 + C_{mix}\delta^2 \sin^2\theta. \tag{S8}$$

The effective coefficients ($B_\theta, A_\theta, \dots$) are algebraically coupled to the primary Landau parameters $(a_i, b_i, \lambda_i, \kappa, \gamma)$ through the defined transformation. Introducing the scaled amplitudes $A_i = a_i\eta_0^2$, $B_i = b_i\eta_0^4$, $\Lambda_L = \lambda_L\eta_0^4$, $K = \kappa\eta_0^4$, and $\Gamma = \gamma\eta_0^4$, where $\eta_0 \approx 0.0315$ Årepresents the physical equilibrium amplitude of the primary L-mode distortion derived from DFT. The explicit mappings are derived:

$$\begin{aligned} C &= 3A_L + 3B_L + 3\Lambda_L \\ B_\theta &= A_M + K + 2\Gamma - A_L - 2B_L - 2\Lambda_L \\ A_\theta &= B_L + B_M - K \\ B_\delta &= 2A_L + 12B_L \\ A_\delta &= 2B_L + \Lambda_L \\ C_{mix} &= 2\Gamma - 2\Lambda_L \end{aligned} \tag{S9}$$

First, consider the chiral rotational coordinate θ. From the mapping relations (Equation S9) and the fitted regression parameters, the effective coefficients are evaluated as $A_\theta \approx -12.2$ meV and $B_\theta \approx 13.5$ meV. The

negative quartic coefficient ($A_\theta < 0$), governed primarily by the large cross-mode repulsion K, ensures an anharmonic drive toward the phase boundary. The positive quadratic curvature ($B_\theta > 0$) establishes the local metastability of the initial $P\bar{3}c1$ configuration ($\theta = 0$). In this rigid-amplitude parameterization ($x = \sin^2\theta$), the polynomial maximum occurs at $x_{max} \approx 0.55$, corresponding to the energetic saddle point along the transition path. While the unrelaxed rigid boundary ($x = 1$) sits higher in energy, subsequent amplitude relaxation ($\eta_M \neq \eta_L$) drops the C_2 ($\theta = \pi/2$) state well below the $P\bar{3}c1$ energy, establishing it as the absolute global minimum. This topology confirms that the strong $L+M$ on-site repulsion (K) creates the distinct double-well potential that fundamentally separates the chiral and achiral phases.

Second, regarding the nematic coordinate δ, the effective harmonic stiffness is governed directly by $B_\delta = 2A_L + 12B_L$. Using the physical amplitude scale, we evaluate this topological stiffness as $B_\delta \approx 14.9$ meV. This positive curvature guarantees that the C_2 phase is a stable minimum rather than a spontaneous instability. Furthermore, the cross-coupling coefficient $C_{mix} = 2(\Gamma - \Lambda_L) \approx 0.16$ meV is effectively negligible, indicating that this nematic softness is almost entirely decoupled from the chiral rotation. We note that this analytical projection accurately captures the physical scale of the nematic softness without requiring higher-order corrections: evaluating the rigid polynomial at the extreme limit $\delta = 0.31$ yields an energetic penalty of $\sim$1.5 meV, which is in agreement with the 1.69 meV derived directly from fully relaxed DFT calculations (Table S8). This quantitative alignment dictates the uniquely shallow topology of the nematic manifold, creating a continuously accessible, sub-meV energy basin for triclinic distortions that unifies the thermodynamic stability of the chiral phase with the dynamic symmetry lowering observed spectroscopically.

S4. Quantum fluctuation along the nematic collective coordinate

S4.1. Construction of the potential and the effective mass

The potential along the nematic coordinate δ was generated starting from the fully triclinic ($\delta = 0.31$, $P1$) structure: an unconstrained structural relaxation of this configuration proceeds continuously back to the $C2$ minimum, with the total energy and the maximum force decreasing monotonically. Single-point energy evaluations at intermediate geometries of this relaxation trajectory yield the values compiled in Table S8. No intervening local minimum is encountered along the path, confirming the single-well topology of the manifold. Since the twofold rotation of $C2$ exchanges the two active L-mode axes ($\delta \to -\delta$), only the $\delta \geq 0$ branch was computed explicitly; the negative branch is its exact symmetry image and the fitted even polynomial enforces this symmetry identically.

An even polynomial $V(\delta) = a\delta^2 + b\delta^4$ was fitted to the data points whose energy difference from the minimum exceeds the numerical noise floor of the calculations (≈ 0.05 meV per $2\times2\times2$ supercell). The adopted coefficients are listed in Table S10; varying the included points changes a by $\lesssim 10\%$. The fitted curvature is compatible with the independent estimate from the Landau analysis of Sec. S3 ($B_\delta \approx 14.9$ meV).

The effective mass of the collective coordinate was extracted from the displacement field between adjacent configurations along the relaxation trajectory,

$$M_{\text{eff}} = \sum_\alpha m_\alpha \left| \frac{d\mathbf{R}_\alpha}{d\delta} \right|^2, \tag{S10}$$

evaluated numerically as the average over consecutive trajectory pairs (pair-to-pair variation $\lesssim 25\%$), giving $M_{\text{eff}} = 3.6 \times 10^{-47}$ kg m^2.

S4.2. Ground state of the one-dimensional coordinate

The one-dimensional Schrödinger equation for δ was solved on a uniform grid spanning $[-0.8, 0.8]$ with hard-wall boundaries; the ground-state density is negligible well inside the box, rendering the results insensitive to the boundary. The ground-state energy is $E_0 = 5.4$ meV, and no excited-state properties enter the conclusions of the present work. The probability density $|\psi_0(\delta)|^2$ (Figure S3) is exactly symmetric, $\langle\delta\rangle = 0$, with $\sqrt{\langle\delta^2\rangle} = 0.26$. Using the proportionality between δ and the intra-trimer bond disproportionation ($\sim$58 mÅ per unit δ; Table S8), the zero-point wavefunction spans configurations with an RMS disproportionation of $\sim$15 mÅ already at $T = 0$. The classical Boltzmann distribution at 10 K is slightly narrower ($\sqrt{\langle\delta^2\rangle} \approx 0.19$), showing that at low temperature the broadening is dominated by quantum zero-point motion rather than thermal population. Notably, the assumed corrugation scale of the well, $\sim$1 meV $\approx k_B \times 10$ K, is reached only at $|\delta| \approx 0.25$, practically coinciding with the width of the zero-point wavefunction itself.

Table S10. Quantities of the one-dimensional quantization of the nematic coordinate δ.

Quantity	Value
Fit coefficients (meV/supercell)	$a = 11.8$, $b = 57.9$
Effective mass M_{eff}	3.6×10^{-47} kg m^2
Characteristic mode energy ω	≈ 6.7 meV (≈ 78 K)
Ground-state energy E_0	5.4 meV
$\langle\delta\rangle$	0 (exact, by symmetry)
$\sqrt{\langle\delta^2\rangle}$ at $T = 0$	0.26 ($\sim$15 mÅ RMS Δd)
$\sqrt{\langle\delta^2\rangle}$ at 10 K (Boltzmann)	0.19 (classical reference)

S4.3. Limitations

The model treats the local δ excursion of a single $2\times2\times2$ supercell within the fixed chiral ($\theta = \pi/2$) sector; it does not describe the chirality-reversal path across the $P\bar{3}c1$ saddle, which requires correlated motion over

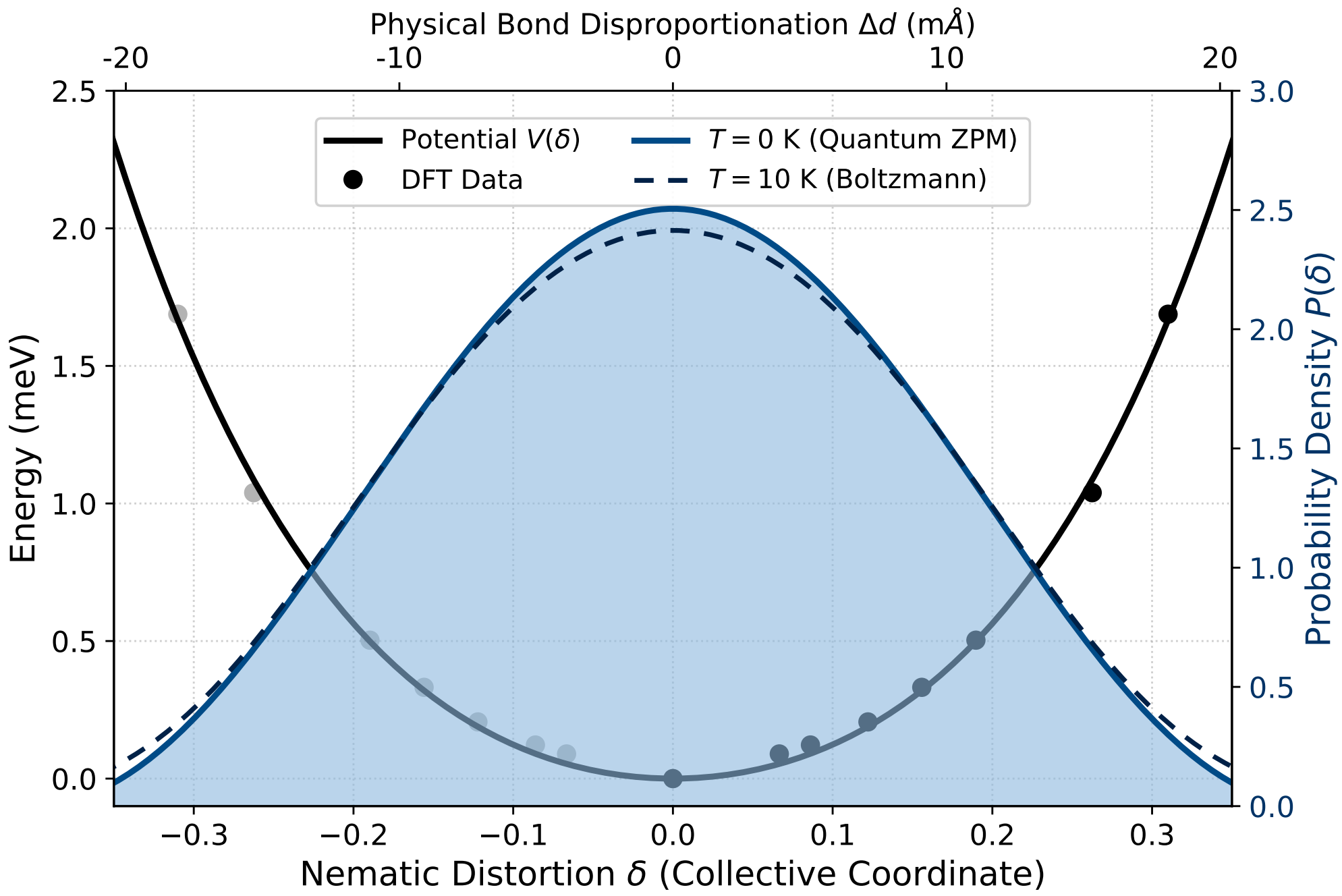

Figure S3. Quantized nematic coordinate on the soft manifold. Black curve: fitted potential $V(\delta) = a\delta^2 + b\delta^4$ ($a = 11.8$, $b = 57.9$ meV per supercell), fitted to the DFT total energies (filled circles) above the numerical-noise floor; hollow circles are the symmetry images at negative δ. Blue curve: ground-state probability density $|\psi_0(\delta)|^2$ ($T = 0$) obtained from the one-dimensional Schrödinger equation with $M_{\text{eff}} = 3.6 \times 10^{-47}$ kg m^2 ($E_0 = 5.4$ meV; right axis). Dashed curve: classical Boltzmann distribution at $T = 10$ K. The ground state is exactly symmetric ($\langle \delta \rangle = 0$) while spanning $\sqrt{\langle \delta^2 \rangle} = 0.26$ (top axis: approximate corresponding intra-trimer bond disproportionation). The potential beyond $|\delta| = 0.31$ is the quartic continuation of the fit.

many supercells. The mutual coupling of the two coordinates is negligible within the fitted Landau functional ($C_{\text{mix}} \approx 0.16$ meV; Sec. S3). The sampled energies follow the relaxation trajectory, which closely approximates the minimum-energy path along this softest structural coordinate. Beyond $|\delta| = 0.31$ the potential is the quartic continuation of the fit; ground-state expectation values vary by $\lesssim 15\%$ under alternative smooth extrapolations.

S5. Band Unfolding and ARPES Simulation

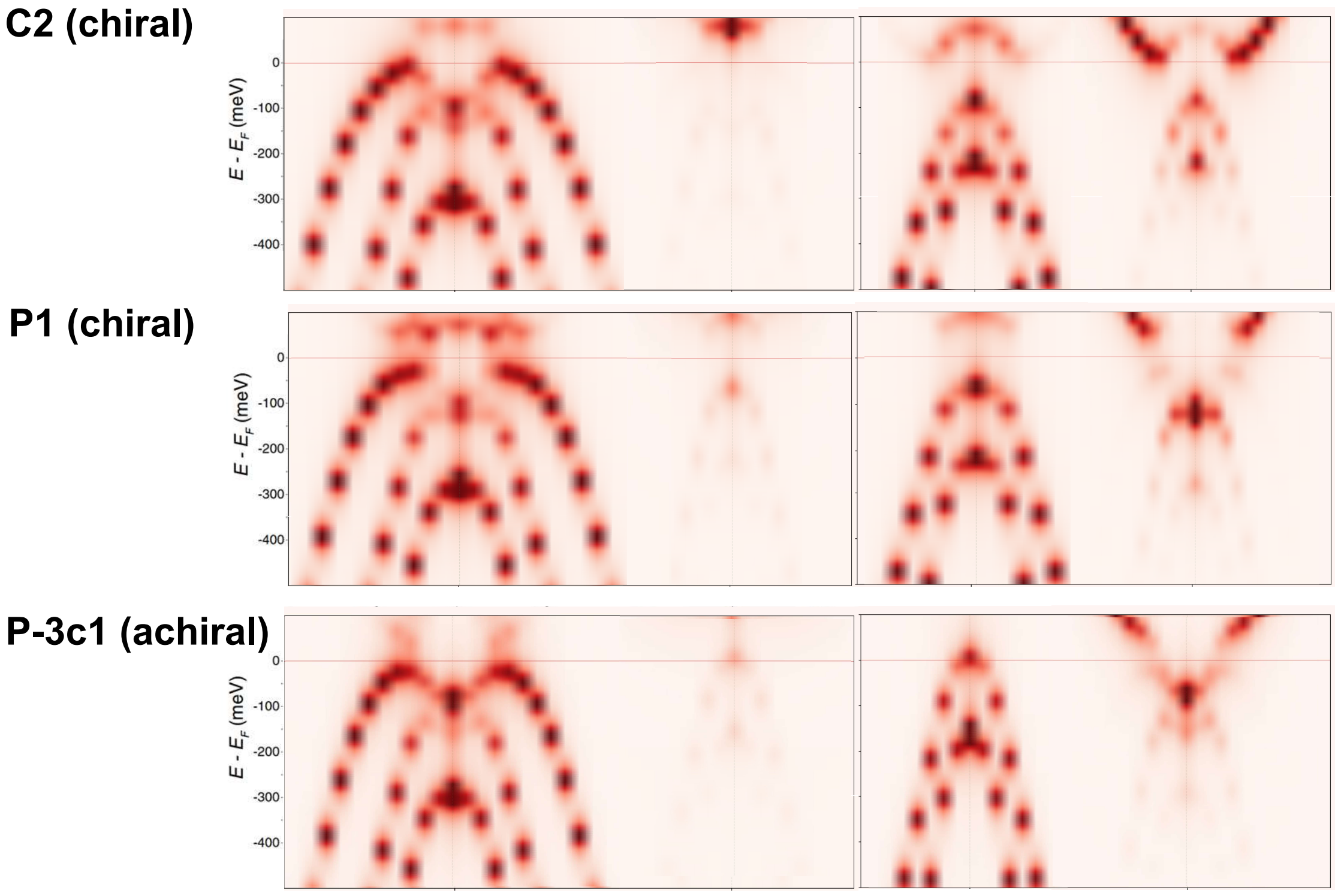

Figure S4. Simulated ARPES spectral functions. (Top) Simulated spectral weight for the chiral $C2$ $(2Q_L + 1Q_M)$ phase. **(Middle)** Simulated spectral weight for the chiral $P1$ $(\delta \sim 0.3)$ phase. **(Bottom)** Simulated spectral weight for the achiral $P\bar{3}c1$ $(3Q_L)$ phase.

*References

[1] John P. Perdew, Kieron Burke,, and Matthias Ernzerhof. “Generalized Gradient Approximation Made Simple.” *Phys. Rev. Lett.*77 (1996): 3865–3868.

[2]

[3] Ji ří Klimeš, David R. Bowler,, and Angelos Michaelides. “Van der Waals density functionals applied to solids.” *Phys. Rev. B*83 (2011): 195131.

[4] James W. Furness, Aaron D. Kaplan, Jinliang Ning, John P. Perdew,, and Jianwei Sun. “Accurate and Numerically Efficient r2SCAN Meta-Generalized Gradient Approximation.” *The Journal of Physical Chemistry Letters* 11, no. 19 (2020): 8208–8215. PMID: 32876454.

[5] Haowei Peng, Zeng-Hui Yang, John P. Perdew,, and Jianwei Sun. “Versatile van der Waals Density Functional Based on a Meta-Generalized Gradient Approximation.” *Phys. Rev. X*6 (2016): 041005.

[6] Li Yin, Hong Tang, Tom Berlijn,, and Adrienn Ruzsinszky. “Efficient simulations of charge density waves in the transition metal Dichalcogenide TiSe2.” *npj Computational Materials* 10, no. 1 (2024): 207.

[7] M. Hellgren, J. Baima, R. Bianco, M. Calandra, F. Mauri,, and L. Wirtz. “Critical role of the exchange interaction for the electronic structure and charge-density-wave formation in $TiSe_2$.” *Phys. Rev. Lett.*119 (2017): 176401.

[8] Kwangrae Kim, Hyun-Woo J. Kim, Seunghyeok Ha, Hoon Kim, Jin-Kwang Kim, Jaehwon Kim, Junyoung Kwon, Jihoon Seol, Saegyeol Jung, Changyoung Kim, Daisuke Ishikawa, Taishun Manjo, Hiroshi Fukui, Alfred Q. R. Baron, Ahmet Alatas, Ayman Said, Michael Merz, Matthieu Le Tacon, Jin Mo Bok, Ki-Seok Kim,, and B. J. Kim. “Origin of the chiral charge density wave in transition-metal dichalcogenide.” *Nature Physics*20 (2024): 1919–1926.
