# Supplementary material for: Chiral‐Nematic Lattice Instability in Charge Density Wave of 1T‐TiSe2
Source: Adv Sci (Weinh). 2026 Oct 20:e78243. Online ahead of print. doi: 10.1002/advs.78243 (PMC13683907; doi:10.1002/advs.78243)
Supplement: Supplementary file 2 [file ADVS-9999-e78243-s001.zip › images/allergy-eps-converted-to.pdf]

Allergy
EUROPEAN JOURNAL OF ALLERGY AND CLINICAL IMMUNOLOGY
EAACI
EUROPEAN ACADEMY OF ALLERGY AND CLINICAL IMMUNOLOGY
