# Supplementary material for: Chiral‐Nematic Lattice Instability in Charge Density Wave of 1T‐TiSe2
Source: Adv Sci (Weinh). 2026 Oct 20:e78243. Online ahead of print. doi: 10.1002/advs.78243 (PMC13683907; doi:10.1002/advs.78243)
Supplement: Supplementary file 2 [file ADVS-9999-e78243-s001.zip › images/1p.pdf]

ORIGINAL ARTICLE

Guidelines for Establishing a Cytometry Laboratory

Anna C. Belkina[1] | Caroline E. Roe[2] | Vera A. Tang[3] | Jessica B. Back[4] | Claudia Bispo[5] | Alexis Conway[6] | Uttara Chakraborty[7] | Kathleen T. Daniels[8] | Gelo de la Cruz[9] | Laura Ferrer-Font[10] | Andrew Filby[11] | David M. Gravano[12] | Michael D. Gregory[13] | Christopher Hall[14] | Christian Kukat[15] | André Mozes[16] | Diana Ordoñez-Rueda[17] | Eva Orlowski-Oliver[18] | Isabella Pesce[19] | Ziv Porat[20] | Nicole J. Poulton[21] | Kristen M. Reifel[22] | Aja M. Rieger[23] | Rachael T. C. Sheridan[24] | Gert Van Isterdael[25] | Rachael V. Walker[14]

Correspondence: Rachael V. Walker (rachael.walker@babraham.ac.uk)

Received: 24 January 2023 | **Revised:** 10 August 2023 | **Accepted:** 3 October 2023

Funding: Bigelow Laboratory for Ocean Sciences; Biotechnology and Biological Sciences Research Council; Canadian Foundation for Innovation, Natural Sciences and Engineering Research Council; Fundação Champalimaud; Hugh Green Foundation; Max Planck Society; National Institutes of Health; Novo Nordisk Fonden; Peter MacCallum Foundation; University of Alberto Faculty of Medicine and Dentistry; University of California Merced Office of Research and Economic Development; University of Newcastle (UK); University of Trento; Van Andel Research Institute; Karmanos Cancer Institute, Grant/Award Number: R50 CA251068-01; The Novo Nordisk Foundation Center for Stem Cell Medicine, reNEW; BBSRC Core Capability Grant; All Authors are past or present ISAC SRL Emerging Leaders

ABSTRACT

The purpose of this document is to provide guidance for establishing and maintaining growth and development of flow cytometry shared resource laboratories. While the best practices offered in this manuscript are not intended to be universal or exhaustive, they do outline key goals that should be prioritized to achieve operational excellence and meet the needs of the scientific community. Additionally, this document provides information on available technologies and software relevant to shared resource laboratories. This manuscript builds on the work of Barsky et al. 2016 published in *Cytometry Part A* and incorporates recent advancements in cytometric technology. A flow cytometer is a specialized piece of technology that require special care and consideration in its housing and operations. As with any scientific equipment, a thorough evaluation of the location, space requirements, auxiliary resources, and support is crucial for successful operation. This comprehensive resource has been written by past and present members of the International Society for Advancement of Cytometry (ISAC) Shared Resource Laboratory (SRL) Emerging Leaders Program https://isac-net.org/general/custom.asp?page=SRL-Emerging-Leaders with extensive expertise in managing flow cytometry SRLs from around the world in different settings including academia and industry. It is intended to assist in establishing a new flow cytometry SRL, re-purposing an existing space into such a facility, or adding a flow cytometer to an individual lab in academia or industry.

Trial Registration: EudraCT number: 2012-005726-30; ClinicalTrials.gov identifier: NCT02171416.

JEL Classification: G11, G12, G14, G18

The pantheon of art is not a timeless present that presents itself to a pure aesthetic consciousness. but the act of a mind and spirit that has collected and gathered itself historically.

(Gadamer, 2013)

1 | Glossary of Terms used in this Resource

To aid comprehension for those new to the field, we have provided a list of specific terms used to describe the different types of commonly used cytometers, as well as a glossary of frequently used cytometric terms and a detailed list of commercially

All authors are current or past ISAC Shared Resource Laboratory Emerging Leaders.

For affiliations, refer to page 31.

This is an open access article under the terms of the Creative Commons Attribution-NonCommercial License, which permits use, distribution and reproduction in any medium, provided the original work is properly cited and is not used for commercial purposes.

©2023 The Authors. *AIChE Journal* published by Wiley Periodicals LLC on behalf of American Institute of Chemical Engineers.
