# Supplementary material for: Chiral‐Nematic Lattice Instability in Charge Density Wave of 1T‐TiSe2
Source: Adv Sci (Weinh). 2026 Oct 20:e78243. Online ahead of print. doi: 10.1002/advs.78243 (PMC13683907; doi:10.1002/advs.78243)
Supplement: Supplementary file 2 [file ADVS-9999-e78243-s001.zip › Figure/Supp3.pdf]

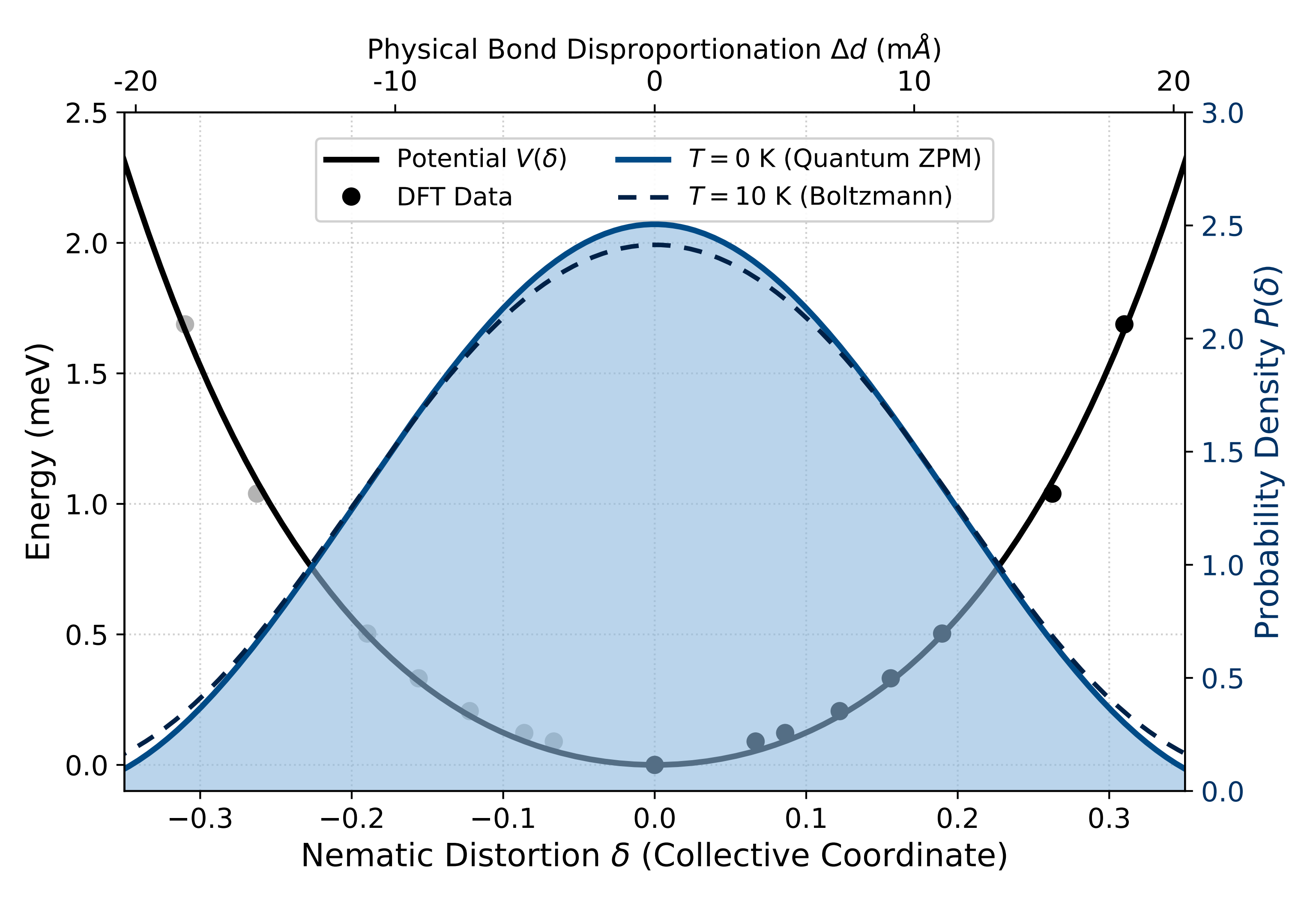

Physical Bond Disproportionation Δd (mÅ)
-20
-10
0
10
20
Potential V(δ)
DFT Data
T = 0 K (Quantum ZPM)
T = 10 K (Boltzmann)
Energy (meV)
2.5
2.0
1.5
1.0
0.5
0.0
Probability Density P(δ)
3.0
2.5
2.0
1.5
1.0
0.5
0.0
−0.3
−0.2
−0.1
0.0
0.1
0.2
0.3
Nematic Distortion δ (Collective Coordinate)
