# Supplementary material for: Chiral‐Nematic Lattice Instability in Charge Density Wave of 1T‐TiSe2
Source: Adv Sci (Weinh). 2026 Oct 20:e78243. Online ahead of print. doi: 10.1002/advs.78243 (PMC13683907; doi:10.1002/advs.78243)
Supplement: Supplementary file 2 [file ADVS-9999-e78243-s001.zip › Figure/Supp2.pdf]

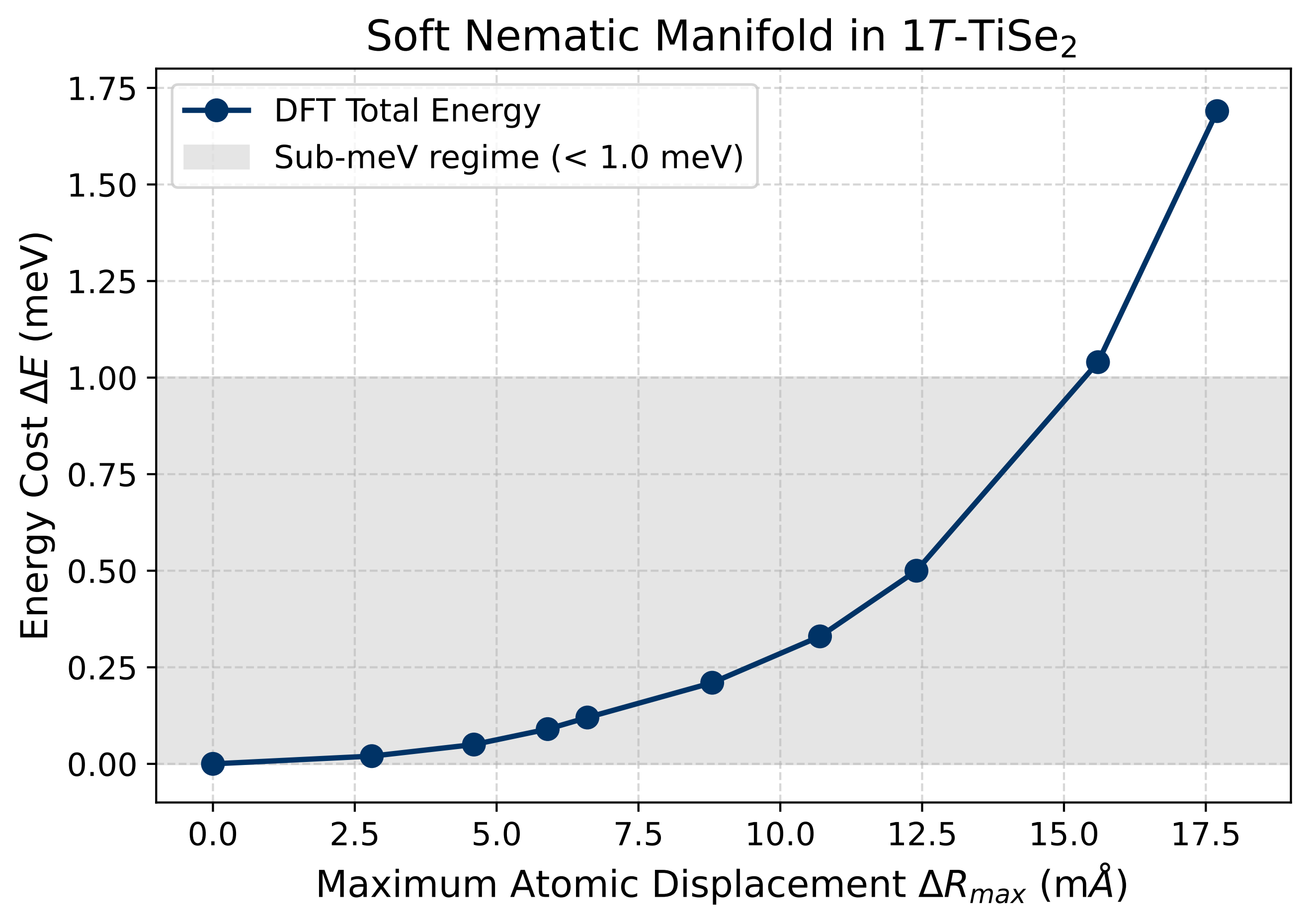

Soft Nematic Manifold in 1T-TiSe2
DFT Total Energy
Sub-meV regime (< 1.0 meV)
Energy Cost ΔE (meV)
Maximum Atomic Displacement ΔR_max (mÅ)
0.00
0.25
0.50
0.75
1.00
1.25
1.50
1.75
0.0
2.5
5.0
7.5
10.0
12.5
15.0
17.5
