# Supplementary material for: Chiral‐Nematic Lattice Instability in Charge Density Wave of 1T‐TiSe2
Source: Adv Sci (Weinh). 2026 Oct 20:e78243. Online ahead of print. doi: 10.1002/advs.78243 (PMC13683907; doi:10.1002/advs.78243)
Supplement: Supplementary file 2 [file ADVS-9999-e78243-s001.zip › Figure/Supp4.pdf]

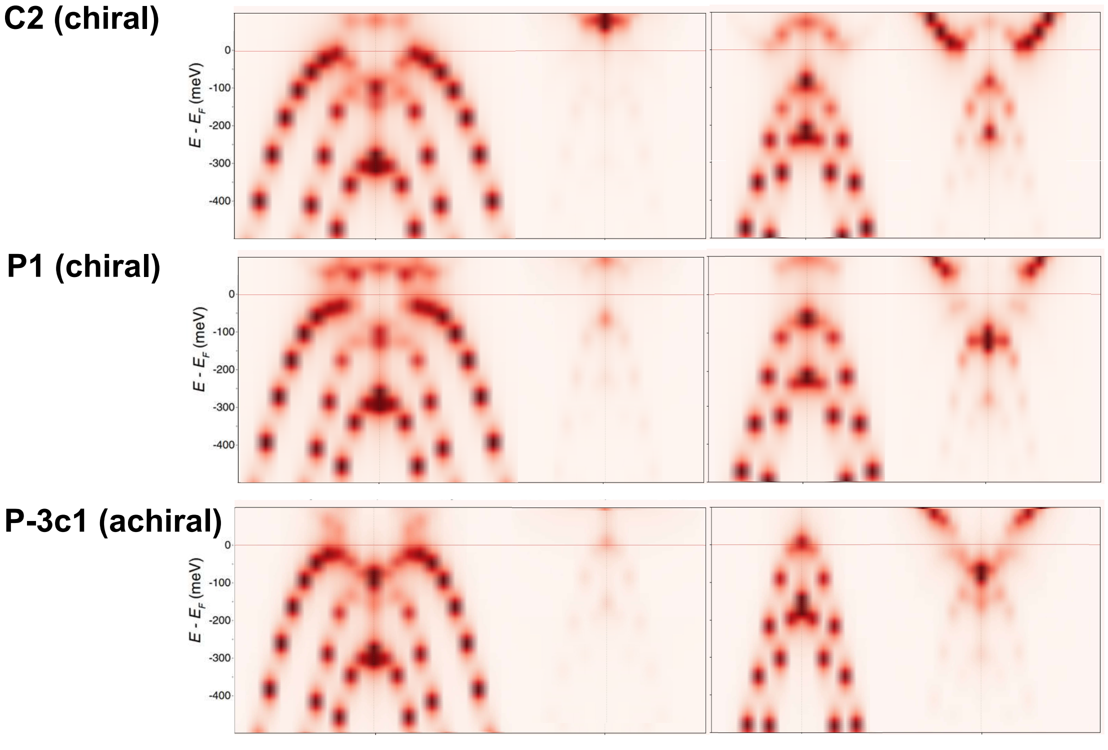
C2 (chiral)
P1 (chiral)
P-3c1 (achiral)
E - E_F (meV)
0
-100
-200
-300
-400
