# Supplementary material for: Chiral‐Nematic Lattice Instability in Charge Density Wave of 1T‐TiSe2
Source: Adv Sci (Weinh). 2026 Oct 20:e78243. Online ahead of print. doi: 10.1002/advs.78243 (PMC13683907; doi:10.1002/advs.78243)
Supplement: Supplementary file 2 [file ADVS-9999-e78243-s001.zip › Figure/Supp1.pdf]

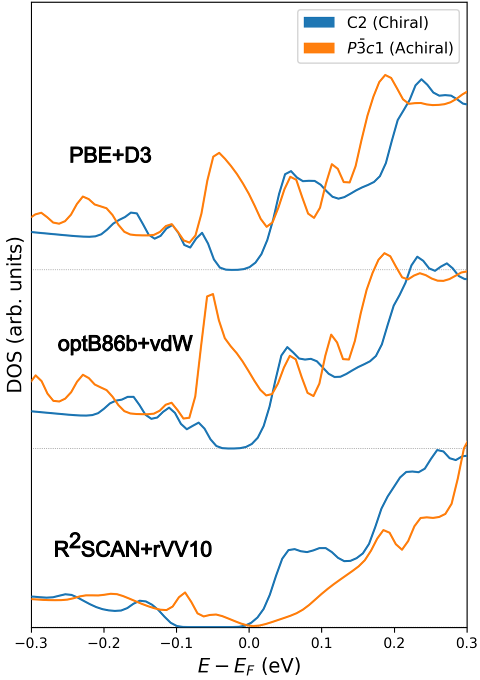

C2 (Chiral)
$P\bar{3}c1$ (Achiral)
PBE+D3
optB86b+vdW
R^2SCAN+rVV10
DOS (arb. units)
−0.3
−0.2
−0.1
0.0
0.1
0.2
0.3
$E - E_F$ (eV)
